# Horizontal gene transfer events reshape the global landscape of arm race between viruses and homo sapiens

**DOI:** 10.1038/srep26934

**Published:** 2016-06-07

**Authors:** Dong-Sheng Chen, Yi-Quan Wu, Wei Zhang, San-Jie Jiang, Shan-Ze Chen

**Affiliations:** 1 Department of Genetics, University of Cambridge, Cambridge, CB2 3EH, UK; 2 Max von Pettenkofer-Institute for Virology, Ludwig-Maximilians-University Munich, 80336 Munich, Germany; 3 Research unit gene vector, Helmholtz Zentrum, 81377 Munich, Germany; 4 Department of Plant Sciences, University of Cambridge, Cambridge, CB2 3EA, UK; 5 Department of Pathophysiology, West China School of Preclinical Sciences and Forensic Medicine, Sichuan University, 610041 Chengdu, Sichuan Province, China

## Abstract

Horizontal gene transfer (HGT) drives the evolution of recipient organism particularly if it provides a novel function which enhances the fitness or its adaption to the environment. Virus-host co-evolution is attractive for studying co-evolutionary processes, since viruses strictly replicate inside of the host cells and thus their evolution is inexorably tangled with host biology. HGT, as a mechanism of co-evolution between human and viruses, has been widely documented, however, the roles HGT play during the interaction between human and viruses are still in their infancy. In this study, we performed a comprehensive analysis on the genes horizontally transferred between viruses and their corresponding human hosts. Our study suggests that the HGT genes in human are predominantly enriched in immune related GO terms while viral HGT genes are tend to be encoded by viruses which promote the invasion of immune system of hosts. Based on our results, it gives us a hint about the evolution trajectory of HGT events. Overall, our study suggests that the HGT between human and viruses are highly relevant to immune interaction and probably reshaped the arm race between hosts and viruses.

Horizontal gene transfer has been well recognized as an important driving force of evolution, as it provides gene flow between evolutionary remote lineages which may facilitate the evolution of recipient spices by providing novel functions^1^,^2^,^3^,^4^,^5^,^6^,^7^,^8^,^9^. Since the first report of HGT from bacteriophage to *corynebacterium diphtheriae* in 1951^10^, many other species varying from prokaryote to eukaryote have also been found to be the recipients of exogenous genes^2^,^5^,^6^,^10^. Most transferred genes are deleterious and will be wiped out by nature selection. In contrast, a small proportion of HGT events provide advantages against selection to hosts and thus can be retained in the species^11^,^12^. For instance, plant-parasitic nematodes gained cellulase genes and thus acquired cellulase activity resulting from HGT from several microbial donors, which significantly enhanced their parasitism and pathogenicity^13^. More interestingly, HGT was found to contribute to the plant parasitic mechanism of oomycetes, as some HGT genes are predicted to metabolize sugars, rutin, hemicellulose for feeding on plant tissues and some HGT genes are predicted to degrade structures components specific to plants which favors its attacking on plant cells^14^. HGT is common in animals as well, for example, a developmentally expressed functional gene in zebrafish was found to be laterally transferred from marine bacteria^15^.

Over the past hundreds of millions of years, viruses and hosts have been undergoing a continuous co-evolutionary process, involving both host defense and viral evasion mechanisms^16^,^17^. Viruses have evolved various strategies to evade host immune system. For instance, herpesviruses, a group of large DNA viruses, have developed to encode proteins that mimic cellular cytokines and cytokine receptors to modulate cytokine mediated signals during infection^18^,^19^,^20^. In contrast, host immune system is due to protect itself from invading pathogens, including viruses. HGT has been frequently reported between hosts and viruses and thus considered as an important contributor for host-virus co-evolution^21^,^22^,^23^,^24^,^25^,^26^,^27^,^28^.

The systematic screen on 26 animal species suggest that there are hundreds of HGT genes in primates, flies and nematodes^29^. Alastair Crisp *et al*. identified hundreds of HGT genes in the genome of human beings which originally transferred from plants, fungus and bacterial^29^. It is of particular interest that they also predicted the HGT which happened between 51 human encoding genes and 27 viral encoding genes. In this study, we investigated the characteristics, evolution history and significance of genes transferred between human and viruses.

## Characterization of the 51 HGT Genes Encoded by Human

**Figure 1:**
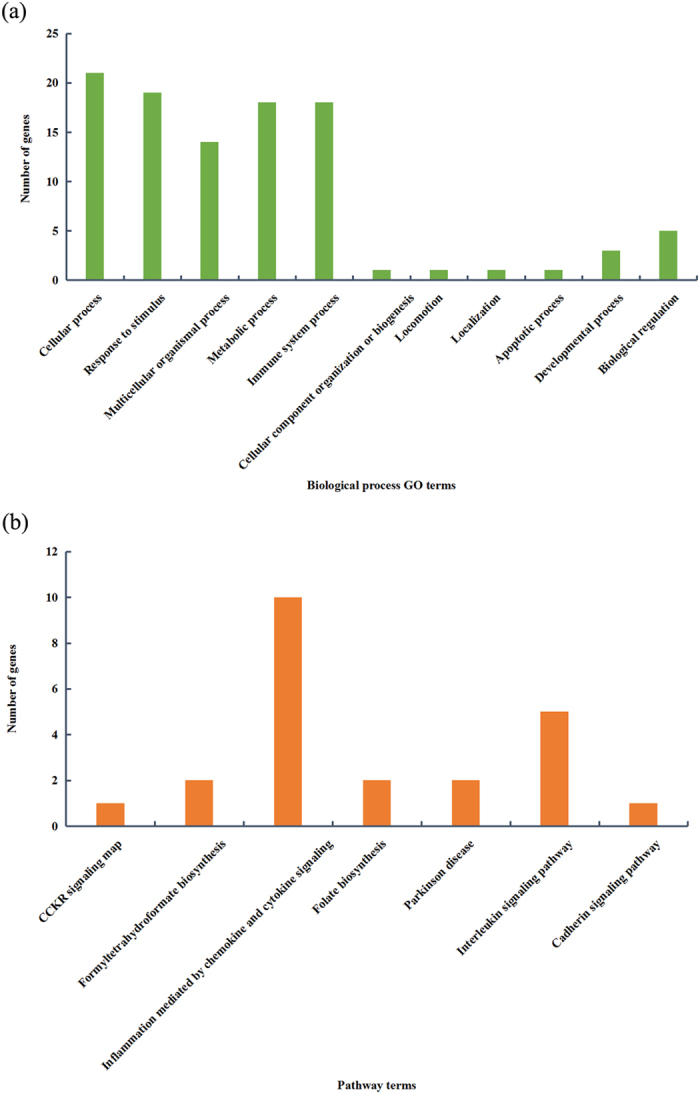
The biological process and pathway classification of the 51 HGT genes encoded by human. (**a**) Biological process classification (**b**) Pathway classification.

In order to study the functions of the 51 HGT genes encoded by human (Supplementary Table S1), the data set was submitted to PANTHER^30^,^31^ for gene list classification analysis. Among the 51 HGT genes, some genes could not be mapped to any GO or pathways terms for lack of annotation while others might be related to multiple terms. Therefore the total number of genes in each classification analysis is not equal to 51 because of overlapping of gene list in different catalogues. Briefly, 27, 29, 5 and 17 genes were successfully mapped in ‘molecular function’, ‘biological process’, ‘celluar process’ and ‘pathway’ terms respectively (Supplementary Table S2). According to the ‘molecular function classification’ result, 27 out of 51 genes were found to have ‘binding activity’(9), ‘receptor activity’(17), ‘catalytic activity’(18) and ‘antioxidant activity’(1). ‘Biological process classification’ suggests that 29 out of 57 genes were mainly mapped to ‘cellular process’, ‘localization’, ‘response to stimulus’, ‘metabolic process’ and ‘immune system process’, with over 18 genes in each catalog (Fig. 1a). In ‘cell components classification analysis’, 5 out of 51 genes were mapped (3 genes encode ‘membrane’, 4 encode ‘cell part’ and 1 encodes ‘organelle’ and the other 1 encodes ‘extracellular region’). Regarding ‘pathway classification’ analysis, 17 out of 51 genes were found to be involved in 7 pathways (Fig. 1b). Specifically, *YES1* from ‘CCKR signalling map pathway’, *Q86XF0* and *P00374* from ‘formyltetrahydroformate biosynthesis pathway’, 10 genes (*CCR3, CCR1, CXCR1, CCR5, CXCR2, CXCR6, CCR6, CCR4, XCR1* and *CCR7*) from ‘inflammation mediated by chemokine and cytokine signalling pathway’, *DHFRL1* and *DHFR* from ‘folate biosynthesis’, *YES1* and *FGR* from ‘parkinson disease pathway’, *CXCR2, CXCR1, IL17A, IL17F, IL10* from ‘Interleukin signalling pathway’, *YES1* from ‘cadherin signalling pathway’. After classification of those HGT genes, we performed a GO enrichment analysis. In total 46 GO terms were found to be enriched with the p value smaller than 0.05 (Supplementary Table S3). Surprisingly, the GO terms predominantly enriched in immune related terms. The most significant GO term is ‘chemokine-mediated signaling pathway’ with a p value 5.70E-18, followed by ‘dendritic cell chemotaxis’ with p value 1.43E-09 and then ‘dendritic cell migration ‘ (p value 7.27E-09). Actually, the top 10 GO terms are all immune related (Table 1). The disproportionate of enriched immune related GO terms strongly suggest that the HGT events between viruses and hosts have a clear link with immune response. When we checked the functional annotation of the 51 gene list, we found 27 genes were found to be directly related to immune response. Specifically, 19 genes encode chemokine receptors, 5 genes encode interleukin(interleukin 1 receptor antagonist, interleukin 1 receptor, type II, interleukin 10, interleukin 17A, interleukin 17F), and 3 genes encode cluster of differentiation (CD) molecules (CD200 molecule,CD59 molecule and CD69 molecule).

### The 27 viral HGT genes are encoded by herpesviridae, poxviridae, retroviridae and bornaviridae

Next, we investigated the origin of the HGT related genes derived from the viral genomes (Supplementary Table S1). The result shows that the 27 viral HGT related genes are found to be encoded by 14 virus species. Specifically, 5 out of 14 species belongs to herpesviridae (human herpesvirus type 5, 6, 8, equine herpesvirus 2, Saimiriine herpesvirus 2), 5 from poxviridae (deerpox virus, molluscum contagiosum virus, vaccinia virus, fowlpox virus, yaba monkey tumor virus), 3 from retroviridae (rous sarcoma virus, baboon endogenous virus, simian retrovirus), with the remaining one from bornaviridae (bornavirus). The common characteristics those viruses share is that they have the capability to establish latent infection, indicating that they are able to modify and evade the immune system of hosts^32^,^33^,^34^,^35^,^36^.

### Orthologs of immune related HGT genes were mainly found in viruses capable of establishing latent infection

To test whether the 27 immune related HGT genes encoded by human have any homologs in other viruses species, we performed a blastp analysis using 27 viral HGT proteins against virus protein database. As the function as of a particular protein is largely determined by its structure, we usually infer the function of an unknown protein based on its similarity to the well-studied protein through homology structure modeling. However, the accuracy of modeling is highly dependent on the sequence identity between two proteins. Models tend to be reliable when sequence identity is above 50%, become less confident in the 30–50% range and show low-accuracy under 30% identity^37^. Therefore, we used two sequence identity cut-off values (30% and 50%) in the ortholog analysis. Our results indicated that there were 1019 records in viruses sharing similarity with the 27 immune related HGT genes encoded by human (Supplementary Table S4). When using a cut-off value of identity greater than 30% and removing the redundant hits, 151 unique viral proteins were left (Supplementary Table S5). We then traced the organisms of those proteins, those 151 viral proteins were mapped to 37 virus families (arteriviridae, asfarviridae, baculoviridae, betaflexiviridae, bromoviridae, bunyaviridae, caliciviridae, caulimoviridae, chrysoviridae, closteroviridae, coronaviridae, flaviviridae, hepadnaviridae, herpesviridae, iridoviridae, malacoherpesviridae, mimiviridae, myoviridae , narnaviridae, ophioviridae, orthomyxoviridae, paramyxoviridae, parvoviridae, phycodnaviridae, picornaviridae, plasmaviridae, podoviridae, poxviridae, reoviridae, retroviridae, rhabdoviridae, siphoviridae, togaviridae, tombusviridae, virgaviridae). Among those, 35 out of the 151 proteins belong to herpesviridae, 18 proteins belong to poxviridae and 11 proteins belong to retroviridae (Supplementary Table S4). In total around 42% (64 out of 151) viral proteins sharing more than 30% identity belong to herpesviridae, poxviridae and retroviridae. Viruses in those three families are well known for their ability of immune evasion and establishment of latent infection. When we increase the cut-off value of identity from 30% to 50%, this trend become even more obvious, only 12 unique viral proteins meet this criteria, and predominantly belongs to herpesviridae (10 out of 12). The remaining two proteins were encoded by retroviridae and bromoviridae respectively (Table 2).

### Similarity between viral encoding G-protein coupled receptor E1 and human encoding chemokine receptors

**Figure 2:**
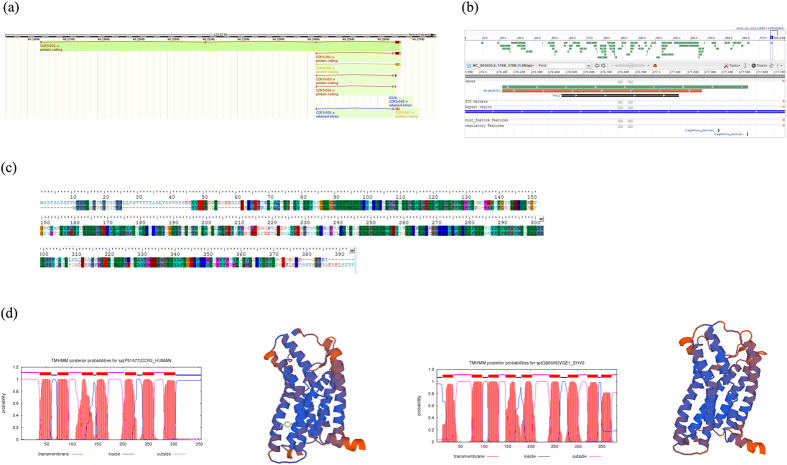
The similarity of protein sequence, secondary structure and 3D structure between human encoding CCR3 and viral protein E1. (**a**) Gene model of human CCR3 (**b**) Gene model of EHV E1 (**c**) Alignment of protein sequences between CCR3 and E1 (**d**) Transmembrane domains and predicted 3D structure of CCR3 and E1.

Of particular interest, we found out that 13 out of the 27 human immune related HGT proteins (chemokine (C-C motif) receptor(CCR) 1–7, CCR9, CCR-like 1 and CCR-like 2, C-X-C motif chemokine receptor 3, 4 and 6) harbor the best hit with the same viral protein, G-protein coupled receptor E1 (Q89609) encoded by equine herpesvirus 2 (EHV-2). The identity between those 13 host proteins and E1 vary from 33.5% to 55.8%. As the identity between CCR3 and E1 is the highest (55.8%), we performed a comparison analysis of protein sequence, transmembrane domains and 3D structure between CCR3 and E1. Overall, CCR3 and E1 exhibit very high degree of similarity. Due to alternative splicing, CCR3 possesses 6 isoforms CCR3-001 (2000bp, 355aa), CCR3-201(1786bp, 376aa), CCR3-002 (1581bp, 355aa), CCR3-007 (1201bp, 355aa), CCR3-003 (400bp, 80aa) and CCR3-004 (212bp, 21aa). E1 is composed of 1149 nucleotides, which is subsequently translated into 383 aa, with 28 more aa than CCR3-001. TMHMM^38^ transmembrane analysis suggests that CCR3 contains 7 transmemembrane domains while E1 contains 9 domains. SWISS MODEL^39^ predicted 3D structures indicate that the two proteins share almost identical structures except for some differences in the terminal regions (Fig. 2).

### Functional divergence after gene duplication contributes to the evolution of CCRs gene family in human

**Figure 3:**
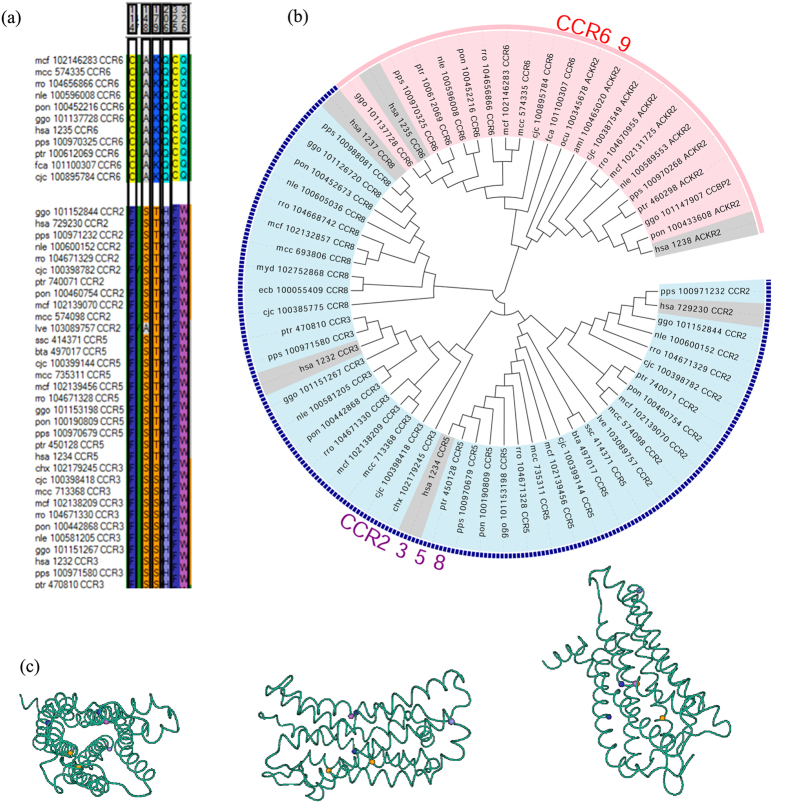
Functional divergence of CCR3s paralogs.

Gene duplication serves as an important source of functional divergence, as duplicated genes may gain novel functions or lose original ones through modifying their existing regulatory network by accumulation of mutations in coding or non-coding regions^40^,^41^. There are 9 copies of C-C chomokine receptors in human genome which share distinct extent of similarity to each other, it is possible that those genes come from functional divergence after gene duplication. Using DIVERGE 3 developed by Gu’s group^42^,^43^, we predicted the type I and type II functional divergence residuals among CCR paralogs. In type I functional divergence, the site is conserved in one group of paralogs while variable in the others. In contrast, type II functional divergence is the case that residuals are conserved in both paralog groups, though the property of amino acids differed between two clusters. Firstly, we constructed a maximum likelihood tree using 66 proteins sequences of CCR 2, 3, 5, 6, 8, 9 downloaded from KEGG. There are two distinct clades on the tree, indicating that all the 66 proteins are divided into two clusters. Cluster A includes CCR6 and CCR9 while cluster B is composed of CCR 2, 3, 5 and 8 (Fig. 3b). We performed the type I and type II functional divergence analysis, which revealed that there was no type I residuals, whereas 6 type II sites were identified at alignment positions 114, 148, 179, 206, 325 and 326 (Fig. 3a). We next studied the property of those amino acids in two clusters and found those residuals change from moderate to hydrophobic, hydrophobic to hydriphibic, positive charge to no charge and hydrophobic to moderate. We also mapped those functional divergence residuals to the 3D structure of CCR3, which supports the deduction that those 5 type II functional divergence residuals contribute to the functional difference between cluster 1 CCRs and cluster 2 CCRs (Fig. 3c).

## Discussion

The gene flow between viruses and hosts normally happens after the integration of viral genomic fragments or whole genomes into host chromosomes. This phenomenon occurs frequently in retroviruses such as human immunodeficiency virus (HIV) through the integration of double stranded DNA (dsDNA) which were reverse transcripted from viral RNA genome^44^,^45^. As for the dsDNA viruses, several members of herpesviruses such as human herpesvirus 6 (HHV-6) and marek’s disease virus (MDV) have been found to be capable to integrate their genomes into host chromosomes under certain circumstances^46^,^47^,^48^. It is possible that herpesviruses integrate into the germ line chromosomes of the ancestor of homo sapiens, probably through non-homologous recombination or interaction with cellular retroelements^49^,^50^. If the immune related genes were horizontally transferred from viruses to host, the foreign genes were likely to be retained in the population for providing protective immunity for recipient hosts. Since then, the laterally transferred genes experienced functional divergence after gene duplication, which subsequently evolved into a family of CCRs with different functions. In our study, we predicted 6 amino acid residuals which might contribute to the functional divergence of CCR paralogs in human, however further research need to be done to validate the functional importance of those residuals. On the other hand, if those foreign genes were transferred from host to viruses, it would be a quite dissimilar story, viruses might acquire genomic fragments containing host origin genes when switching from latency to lytic infection.

**Figure 4:**
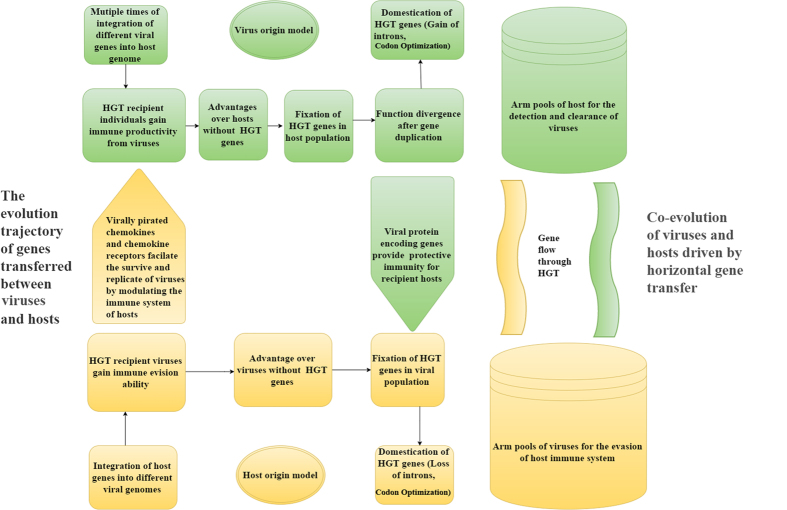
Horizontal gene transfer drives the co-evolution of hosts and viruses.

In a recent report by Crisp *et al*., the direction of gene transfer between viruses and humans remains an open question^29^. In our opinion, there are two possible models to explain the HGT direction, the ‘host origin model’ in which those HGT genes were laterally transferred from host to viruses, and the ‘virus origin model’ in which those HGT genes were transferred in an opposite direction. In theory, these two scenarios could happen with equal chance. In the former scenario, namely ‘host origin model’, the integration of viral structural gene into host genome could lead to the expression of viral antigen which would stimulate the immune system to protect itself against the infection of viruses. In other words, host might utilize the viral DNA as ‘DNA vaccine’ by acquiring and expressing viral protein encoding genes^51^,^52^. However, the HGT genes are unlikely to be fixed in host population unless they meet two criteria. Firstly, the HGT genes need to be integrated into the chromosome of sperms, otherwise the HGT will be difficult to be inherited by offsprings of the HGT recipient individuals. Secondly, the HGT genes should provide enhanced fitness to the environment compared to counterparts without the transfer of foreign genes. Once the viral origin HGT genes got spread and fixed in the population, they probably experienced domestication such as the optimizing of codons and the gain of introns. During millions of years, those HGT genes evolved into a family of proteins playing diverse roles in immune system. In contrast, in the second scenario (‘host origin model’), those immune related factors might be used by viruses to trick the immune response of hosts to achieve the goal of immune evasion. Viruses have been reported to hijack host immune response related signaling pathways by mimicking host immune modifiers, either cytokines or chemokines, in order to facilitate the replicate and survive of viruses insides host cells^53^,^54^,^55^,^56^,^57^,^58^,^59^,^60^. In our study, among the 51 human encoding HGT genes transferred between human and viruses, several immune related genes were found to share high similarity with viral proteins encoded by herpesviridae, poxviridae and retroviridae. As a variety of viruses have been shown to restrict the immune system of the host by protein imitating the host immune functional molecular^61^,^62^, it is highly possible that those viruses investigated in this study utilize a similar mechanism to tackle with the host immune system. Viruses carrying genes related to immune response might facilitate them to evade the immune system, which enhances their opportunity to survive the attack from host immune system and pass on their genetic elements to progeny viruses. Taken together, ‘host origin model’ and ‘virus origin model’ are not necessarily mutually exclusive. It could be possible that some genes were transferred from hosts to viruses while others from viruses to hosts. And it is the bi-directional gene flow of immune related genes through HGT that drives the co-evolution of viruses and hosts and altered the attack and defense strategies of players during the lasting war among the two sides (Fig. 4).

The comparison analysis suggests a high similarity of both secondary structure and 3D structure between viral protein E1 and host protein CCR3, implying they share similar biological functions as well. As CCR3 is well known for its importance in inducing the migration of immune cells into inflammatory sites^63^,^64^,^65^, it is reasonable to speculate that viral protein E1 is also related to immune response. Since the particular mechanism by which E1 manipulate host immune system is not yet fully understood, more work needs to be done to clarify the roles of E1 in the interaction between viruses and their hosts. Intriguingly, the 51 HGT genes in human are enriched in immune related GO terms, which suggests the HGT events are tend to happen among immune genes. In addition, the 27 viral HGT genes are encoded by viruses from herpesviridae, retroviridae and bornaviridae, which are capable to establish latent infection, suggesting HGT might contribute to the immune evasion of those viral species. In conclusion, our study suggests that the HGT between viruses and hosts might play a pivotal role in reshaping the arm race between viruses and hosts, which eventually drives their co-evolution.

## Methods

### Classification and GO enrichment analysis of gene lists

The genes lists were analysed using PANTHER^31^ classification tool with the default parameters. The GO enrichment analysis was performed using online tool of Gene Ontology Consortium^66^. Specifically, PANTHER overrepresentation test (release 20150430) with Bonferroni correction and GO ontology database (released 2015-08-06) were used to evaluate the enrichment of GO terms for the 51 human HGT genes, all the GO terms with P value greater than 0.05 were removed.

### Blast of 27 human encoding immune related genes

The virus protein database were created using makeblast command from blast package, using the virus sequences downloaded from Uniprot^67^. Blastp programme was used to scan homologs for query protein sequences. Then 27 human immune related HGT protein sequences were blasted against the virus protein databases. The Blastp results were outputted as table format using the parameter outfmt 6. The list of Blastp hit records were extracted for further analysis. Unique viral protein Id were extracted by removing the redundant contents. To investigate the origin of those proteins, the protein IDs were submitted to UniProt using retrieve/ID mapping tool.

### Transmemembrane domains prediction

The transmembrane protein topology of E1 and CCR3 were predicted with TMHMM^38^ while 3D structures were modelled using SWISS MODEL^39^ with default parameters.

### Functional divergence analysis of CCRs genes

The orthlogs of human CCRs were downloaded from KEGG database and analysed using DIVERGE 3^42^,^43^.

## Additional Information

**How to cite this article**: Chen, D.S. *et al*. Horizontal gene transfer events reshape the global landscape of arm race between viruses and homo sapiens. *Sci. Rep.* **6**, 26934; doi: 10.1038/srep26934 (2016).

## Supplementary Material

Supplementary Information

## Figures and Tables

**Table 1:** The top 10 GO enrichment of the 51 human encoded HGT genes.

**GO biological process**	**P value**
Chemokine-Mediated Signaling Pathway (GO:0070098)	5.70E-18
Dendritic Cell Chemotaxis (GO:0002407)	1.43E-09
Dendritic Cell Migration (GO:0036336)	7.27E-09
Inflammatory Response (GO:0006954)	7.62E-09
Chemotaxis (GO:0006935)	2.05E-08
Taxis (GO:0042330)	2.05E-08
Cellular Response To Cytokine Stimulus (GO:0071345)	2.93E-08
Cytokine-Mediated Signaling Pathway (GO:0019221)	3.06E-08
Response To Cytokine (GO:0034097)	1.75E-07
Defense Response (GO:0006952)	8.64E-07

**Table 2:** List of viral genes sharing similarity (identity > = 50%) to 27 human immune related HGT genes.

**Entry**	**Gene names**	**Taxonomic lineage (FAMILY)**
Q8UYT3	ORF2b	Bromoviridae
Q00996	15	Herpesviridae
P04048	V-KIT	Retroviridae
Q89609	E1	Herpesviridae
Q69140	EBNA6 BERF3-BERF4	Herpesviridae
P68677	E7	Herpesviridae
P68678	E7	Herpesviridae
P0CAP9	BCRF1	Herpesviridae
P03180	BCRF1	Herpesviridae
P0C6Z6	BCRF1	Herpesviridae
P24916	13 KCLF2	Herpesviridae
O40633	13	Herpesviridae
