# Supplementary material for: Horizontal gene transfer events reshape the global landscape of arm race between viruses and homo sapiens
Source: Sci Rep. 2016 Jun 7;6:26934. doi: 10.1038/srep26934 (PMC4895215; doi:10.1038/srep26934)
Supplement: Supplementary Information [file srep26934-s1.pdf]

Horizontal gene transfer events reshape the global landscape of arm race between viruses and homo sapiens

Dong-Sheng Chen, Yi-Quan Wu, Wei Zhang, San-Jie Jiang, and Shan-Ze Chen

Supplementary Table S1. List of genes transferred between viruses and human 2

Supplementary Table S2. Functional classification analysis of the 51 human HGT genes 5

Supplementary Table S3. List of enriched GO terms for the 51 human HGT genes 8

Supplementary Table S4. List 1019 BLASTP results for 27 human immune proteins 9

Supplementary Table S5. List of 151 viral proteins share similarity higher then 30% with 27 human immune related proteins 73

Supplementary Table S1. List of genes transferred between viruses and human

Human gene ID	Human gene name	Virus gene ID	Virus gene names	Virus organism
ENSG00000000938	Gardner-Rasheed feline sarcoma viral (v-fgr) oncogene homolog	P00526	V-SRC	Rous sarcoma virus (strain Prague C) (RSV-PrC)
ENSG00000010810	FYN oncogene related to SRC, FGR, YES	P00526	V-SRC	Rous sarcoma virus (strain Prague C) (RSV-PrC)
ENSG00000069493	C-type lectin domain family 2, member D	Q08FK8	DpV83gp144	Deerpox virus (strain Mule deer/United States/W-848-83/1983) (DPV)
ENSG00000085063	CD59 molecule, complement regulatory protein	Q00996	15	Saimiriine herpesvirus 2 (strain 11) (SaHV-2) (Herpesvirus saimiri)
ENSG00000091972	CD200 molecule	P0C788	K14	Human herpesvirus 8 type P (isolate GK18) (HHV-8) (Kaposi's sarcoma-associated herpesvirus)
ENSG00000099377	hydroxy-delta-5-steroid dehydrogenase, 3 beta- and steroid delta-isomerase 7	Q98318	MC152R H-M-N-7	Molluscum contagiosum virus subtype 1 (MOCV) (MCVI)
ENSG00000106366	serpin peptidase inhibitor, clade E (nexin, plasminogen activator inhibitor type 1), member 1	Q08FY2	DpV83gp018	Deerpox virus (strain Mule deer/United States/W-848-83/1983) (DPV)
ENSG00000110080	ST3 beta-galactoside alpha-2,3-sialyltransferase 4	Q08FK2	DpV83gp150	Deerpox virus (strain Mule deer/United States/W-848-83/1983) (DPV)
ENSG00000110848	CD69 molecule	Q08FK8	DpV83gp144	Deerpox virus (strain Mule deer/United States/W-848-83/1983) (DPV)
ENSG00000112115	interleukin 17A	P24916	13 KCLF2	Saimiriine herpesvirus 2 (strain 11) (SaHV-2) (Herpesvirus saimiri)
ENSG00000112116	interleukin 17F	P24916	13 KCLF2	Saimiriine herpesvirus 2 (strain 11) (SaHV-2)

				(Herpesvirus saimiri)
ENSG00000112486	chemokine (C-C motif) receptor 6	Q89609	E1	Equine herpesvirus 2 (strain 86/87) (EHV-2)
ENSG00000115590	interleukin 1 receptor, type II	P25212	VACWR197 B15R	Vaccinia virus (strain Western Reserve) (VACV) (Vaccinia virus (strain WR))
ENSG00000118402	ELOVL fatty acid elongase 4	Q9J5F5	FPV048	Fowlpox virus (strain NVSL) (FPV)
ENSG00000121797	chemokine (C-C motif) receptor-like 2	Q89609	E1	Equine herpesvirus 2 (strain 86/87) (EHV-2)
ENSG00000121807	chemokine (C-C motif) receptor 2	Q89609	E1	Equine herpesvirus 2 (strain 86/87) (EHV-2)
ENSG00000121966	chemokine (C-X-C motif) receptor 4	Q89609	E1	Equine herpesvirus 2 (strain 86/87) (EHV-2)
ENSG00000126353	chemokine (C-C motif) receptor 7	Q89609	E1	Equine herpesvirus 2 (strain 86/87) (EHV-2)
ENSG00000129048	chemokine (C-C motif) receptor-like 1	Q89609	E1	Equine herpesvirus 2 (strain 86/87) (EHV-2)
ENSG00000134259	nerve growth factor (beta polypeptide)	Q9J5D9	FPV072	Fowlpox virus (strain NVSL) (FPV)
ENSG00000136634	interleukin 10	P68678	E7	Equine herpesvirus 2 (strain 86/87) (EHV-2)
ENSG00000136689	interleukin 1 receptor antagonist	Q08FU8	DpV83gp054	Deerpox virus (strain Mule deer/United States/W-848-83/1983) (DPV)
ENSG00000144648	chemokine binding protein 2	Q6TV00	7L	Yaba monkey tumor virus (strain VR587) (YMTV)
ENSG00000160791	chemokine (C-C motif) receptor 5 (gene/pseudogene)	Q89609	E1	Equine herpesvirus 2 (strain 86/87) (EHV-2)
ENSG00000163464	chemokine (C-X-C motif) receptor 1	Q6TV00	7L	Yaba monkey tumor virus (strain VR587) (YMTV)
ENSG00000163823	chemokine (C-C motif) receptor 1	Q89609	E1	Equine herpesvirus 2 (strain 86/87) (EHV-2)
ENSG00000168329	chemokine (C-X3-C motif) receptor 1	D3YS56	US28	Human cytomegalovirus (strain Toledo) (HHV-5) (Human herpesvirus 5)
ENSG00000169508	G protein-coupled receptor 183	Q9J529	FPV206	Fowlpox virus (strain NVSL) (FPV)
ENSG00000172215	chemokine (C-X-C motif) receptor 6	Q89609	E1	Equine herpesvirus 2 (strain 86/87) (EHV-2)
ENSG00000173578	chemokine (C motif) receptor 1	Q89609	E1	Equine herpesvirus 2 (strain 86/87) (EHV-2)

ENSG00000173585	chemokine (C-C motif) receptor 9	Q89609	E1	Equine herpesvirus 2 (strain 86/87) (EHV-2)
ENSG00000176105	v-yes-1 Yamaguchi sarcoma viral oncogene homolog 1	P00526	V-SRC	Rous sarcoma virus (strain Prague C) (RSV-PrC)
ENSG00000176153	glutathione peroxidase 2 (gastrointestinal)	Q98234	GPX1 MC066L	Molluscum contagiosum virus subtype 1 (MOCV) (MCVI)
ENSG00000178409	BEN domain containing 3	Q98204	MC036R	Molluscum contagiosum virus subtype 1 (MOCV) (MCVI)
ENSG00000178700	dihydrofolate reductase-like 1	P09503	DHFR 2	Saimiriine herpesvirus 2 (strain 11) (SaHV-2) (Herpesvirus saimiri)
ENSG00000179934	chemokine (C-C motif) receptor 8	Q6TV00	7L	Yaba monkey tumor virus (strain VR587) (YMTV)
ENSG00000180871	chemokine (C-X-C motif) receptor 2	Q6TV00	7L	Yaba monkey tumor virus (strain VR587) (YMTV)
ENSG00000183597	C22orf25	Q9J5E1	FPV070	Fowlpox virus (strain NVSL) (FPV)
ENSG00000183625	chemokine (C-C motif) receptor 3	Q89609	E1	Equine herpesvirus 2 (strain 86/87) (EHV-2)
ENSG00000183671	G protein-coupled receptor 1	Q9J5I0	FPV021	Fowlpox virus (strain NVSL) (FPV)
ENSG00000183813	chemokine (C-C motif) receptor 4	Q89609	E1	Equine herpesvirus 2 (strain 86/87) (EHV-2)
ENSG00000186810	chemokine (C-X-C motif) receptor 3	Q89609	E1	Equine herpesvirus 2 (strain 86/87) (EHV-2)
ENSG00000188211	natural killer cell cytotoxicity receptor 3 ligand 1	P10272	pol	Baboon endogenous virus (strain M7)
ENSG00000188393	C-type lectin domain family 2, member A	Q08FK8	DpV83gp144	Deerpox virus (strain Mule deer/United States/W-848-83/1983) (DPV)
ENSG00000197122	v-src sarcoma (Schmidt-Ruppin A-2) viral oncogene homolog (avian)	P00526	V-SRC	Rous sarcoma virus (strain Prague C) (RSV-PrC)
ENSG00000228716	dihydrofolate reductase	P09503	DHFR 2	Saimiriine herpesvirus 2 (strain 11) (SaHV-2) (Herpesvirus saimiri)
ENSG00000233276	glutathione peroxidase 1	Q98234	GPX1 MC066L	Molluscum contagiosum virus subtype 1 (MOCV) (MCVI)
ENSG00000235878	AP001468.1	Q69566	U88	Human herpesvirus 6A (strain Uganda-1102) (HHV-6

				variant A) (Human B lymphotropic virus)
ENSG00000242950	ERVW-1	P10269	env	Baboon endogenous virus (strain M7)
ENSG00000244476	ERVFRD-1	P51515	env	Simian retrovirus SRV-2
ENSG00000255423	endogenous Bornavirus-like nucleoprotein 2	P0C797	N	Borna disease virus (strain V) (BDV)

Supplementary Table S2. Functional classification analysis of the 51 human HGT genes

Biological process term	Number of genes	Gene ID
cellular process (GO:0009987)	21	ENSG00000112116 ENSG00000136634 ENSG00000179934 ENSG00000176105 ENSG00000112115 ENSG00000134259 ENSG00000144648 ENSG00000163823 ENSG00000085063 ENSG00000163464 ENSG00000000938 ENSG00000160791 ENSG00000180871 ENSG00000183671 ENSG00000129048 ENSG00000172215 ENSG00000112486 ENSG00000183813 ENSG00000173578 ENSG00000126353 ENSG00000169508
response to stimulus (GO:0050896)	19	ENSG00000136634 ENSG00000176105 ENSG00000134259 ENSG00000144648 ENSG00000069493 ENSG00000163464 ENSG00000000938 ENSG00000160791 ENSG00000180871 ENSG00000110848 ENSG00000183671 ENSG00000129048 ENSG00000172215 ENSG00000112486 ENSG00000188211 ENSG00000183813 ENSG00000173578 ENSG00000126353 ENSG00000176153
multicellular organismal process (GO:0032501)	14	ENSG00000179934 ENSG00000134259 ENSG00000144648 ENSG00000163823 ENSG00000163464 ENSG00000160791 ENSG00000180871 ENSG00000183671 ENSG00000129048 ENSG00000172215 ENSG00000112486 ENSG00000183813 ENSG00000173578 ENSG00000126353
metabolic process (GO:0008152)	18	ENSG00000179934 ENSG00000176105 ENSG00000144648 ENSG00000163823 ENSG00000163464 ENSG00000000938 ENSG00000160791 ENSG00000180871 ENSG00000118402 ENSG00000129048 ENSG00000172215 ENSG00000178700

		ENSG00000112486 ENSG00000183813 ENSG00000173578 ENSG00000228716 ENSG00000126353 ENSG00000176153
immune system process (GO:0002376)	18	ENSG00000112116 ENSG00000136634 ENSG00000176105 ENSG00000112115 ENSG00000144648 ENSG00000069493 ENSG00000163464 ENSG00000000938 ENSG00000160791 ENSG00000180871 ENSG00000129048 ENSG00000172215 ENSG00000112486 ENSG00000188211 ENSG00000183813 ENSG00000173578 ENSG00000126353 ENSG00000176153
cellular component organization or biogenesis (GO:0071840)	1	ENSG00000134259
locomotion (GO:0040011)	1	ENSG00000000938
localization (GO:0051179)	1	ENSG00000000938
apoptotic process (GO:0006915)	1	ENSG00000134259
developmental process (GO:0032502)	3	ENSG00000176105 ENSG00000134259 ENSG00000000938
biological regulation (GO:0065007)	5	ENSG00000136634 ENSG00000176105 ENSG00000134259 ENSG00000000938 ENSG00000183671

**Molecular function terms**	**Number of genes**	**Gene ID**
binding (GO:0005488)	9	ENSG00000112116 ENSG00000136634 ENSG00000176105 ENSG00000112115 ENSG00000134259 ENSG00000069493 ENSG00000000938 ENSG00000110848 ENSG00000183671
receptor activity (GO:0004872)	17	ENSG00000179934 ENSG00000144648 ENSG00000069493 ENSG00000163823 ENSG00000163464 ENSG00000160791 ENSG00000180871 ENSG00000110848 ENSG00000183671 ENSG00000129048 ENSG00000172215 ENSG00000112486 ENSG00000188211 ENSG00000183813 ENSG00000173578 ENSG00000126353 ENSG00000169508
catalytic activity (GO:0003824)	18	ENSG00000179934 ENSG00000176105 ENSG00000144648 ENSG00000163823 ENSG00000163464 ENSG00000000938 ENSG00000160791 ENSG00000180871 ENSG00000118402 ENSG00000129048 ENSG00000172215 ENSG00000178700

		ENSG00000112486 ENSG00000183813 ENSG00000173578 ENSG00000228716 ENSG00000126353 ENSG00000176153

**Cell components terms**	**Number of genes**	**Gene ID**
membrane (GO:0016020)	3	ENSG00000176105 ENSG00000000938 ENSG00000183671
cell part (GO:0044464)	4	ENSG00000176105 ENSG00000134259 ENSG00000000938 ENSG00000183671
organelle (GO:0043226)	1	ENSG00000134259
extracellular region (GO:0005576)	1	ENSG00000136634

**Pathway terms**	**Number of genes**	**Gene ID**
CCKR signaling map (P06959)	1	ENSG00000176105
Formyltetrahydroformate biosynthesis (P02743)	2	ENSG00000178700 ENSG00000228716
Inflammation mediated by chemokine and cytokine signaling pathway (P00031)	10	ENSG00000179934 ENSG00000163823 ENSG00000163464 ENSG00000160791 ENSG00000180871 ENSG00000172215 ENSG00000112486 ENSG00000183813 ENSG00000173578 ENSG00000126353
Folate biosynthesis (P02742)	2	ENSG00000178700 ENSG00000228716
Parkinson disease (P00049)	2	ENSG00000176105 ENSG00000000938
Interleukin signaling pathway (P00036)	5	ENSG00000112116 ENSG00000136634 ENSG00000112115 ENSG00000163464 ENSG00000180871
Cadherin signaling pathway (P00012)	1	ENSG00000176105

Supplementary Table S3. List of enriched GO terms for the 51 human HGT genes

GO biological process	P-value
chemokine-mediated signaling pathway (GO:0070098)	5.70E-18
dendritic cell chemotaxis (GO:0002407)	1.43E-09
dendritic cell migration (GO:0036336)	7.27E-09
inflammatory response (GO:0006954)	7.62E-09
chemotaxis (GO:0006935)	2.05E-08
taxis (GO:0042330)	2.05E-08
cellular response to cytokine stimulus (GO:0071345)	2.93E-08
cytokine-mediated signaling pathway (GO:0019221)	3.06E-08
response to cytokine (GO:0034097)	1.75E-07
defense response (GO:0006952)	8.64E-07
leukocyte chemotaxis (GO:0030595)	3.64E-06
cellular response to organic substance (GO:0071310)	4.53E-06
G-protein coupled receptor signaling pathway (GO:0007186)	5.89E-06
cellular response to chemical stimulus (GO:0070887)	9.72E-06
cell surface receptor signaling pathway (GO:0007166)	2.03E-05
locomotion (GO:0040011)	2.89E-05
leukocyte migration (GO:0050900)	3.29E-05
positive regulation of cytosolic calcium ion concentration (GO:0007204)	3.36E-05
cell chemotaxis (GO:0060326)	4.15E-05
response to organic substance (GO:0010033)	4.32E-05
immune response (GO:0006955)	5.82E-05
response to external stimulus (GO:0009605)	9.57E-05
cytosolic calcium ion homeostasis (GO:0051480)	1.13E-04
immune system process (GO:0002376)	1.67E-04
response to chemical (GO:0042221)	6.43E-04

cellular response to stimulus (GO:0051716)	1.45E-03
cellular calcium ion homeostasis (GO:0006874)	1.71E-03
single organism signaling (GO:0044700)	1.79E-03
signaling (GO:0023052)	1.81E-03
calcium ion homeostasis (GO:0055074)	2.36E-03
cellular divalent inorganic cation homeostasis (GO:0072503)	2.36E-03
response to stimulus (GO:0050896)	2.36E-03
cell communication (GO:0007154)	2.89E-03
divalent inorganic cation homeostasis (GO:0072507)	3.79E-03
response to stress (GO:0006950)	7.10E-03
cell migration (GO:0016477)	1.16E-02
cellular metal ion homeostasis (GO:0006875)	1.61E-02
signal transduction (GO:0007165)	1.65E-02
localization of cell (GO:0051674)	2.50E-02
cell motility (GO:0048870)	2.50E-02
cellular cation homeostasis (GO:0030003)	2.88E-02
cellular ion homeostasis (GO:0006873)	3.34E-02
interleukin-8-mediated signaling pathway (GO:0038112)	3.55E-02
cellular response to interleukin-8 (GO:0098759)	3.55E-02
response to interleukin-8 (GO:0098758)	3.55E-02
metal ion homeostasis (GO:0055065)	4.77E-02

Supplementary Table S4. List 1019 BLASTP results for 27 human immune proteins

query sequence id	subject sequenc	percenta ge of	alignme nt	number of mismatch	number of gap	start of alignme	end of alignme	start of alignme	end of alignme	evalu e	bit scor

	e id	identical matches	length	es	openin gs	nt in query	nt in query	nt in subject	nt in subject		e
tr|F8W7G1|F8W7G1_HUMAN	P0C788	37.14	140	88	0	1	140	63	202	4.00E-27	104
tr|F8W7G1|F8W7G1_HUMAN	Q89738	26.32	171	98	5	41	184	119	288	2.00E-08	52.4
tr|F8W7G1|F8W7G1_HUMAN	Q9QJ17	37.29	59	36	1	41	99	120	177	1.00E-06	46.6
tr|F8W7G1|F8W7G1_HUMAN	P0CW72	25.51	98	62	4	42	130	99	194	0.84	29.3
tr|F8W7G1|F8W7G1_HUMAN	P03228	25.51	98	62	4	42	130	99	194	0.84	29.3
tr|F8W7G1|F8W7G1_HUMAN	P0C6N0	25.51	98	62	4	42	130	99	194	0.84	29.3
tr|F8W7G1|F8W7G1_HUMAN	P35934	46.67	30	13	2	92	118	275	304	4.6	27.3
tr|F8W7G1|F8W7G1_HUMAN	P00545	26.37	91	63	2	16	103	379	468	9.3	26.2
tr|F8W7G1|F8W7G1_HUMAN	Q5GA87	36.21	58	35	2	80	136	173	229	9.7	25.8
tr|F8WC99|F8WC99_HUMAN	P0C788	34.9	149	85	2	1	149	1	137	1.00E-25	99.4
tr|F8WC99|F8WC99_HUMAN	P33807	28.75	80	50	4	47	123	32	107	0.24	30.4
tr|F8WC99|F8WC99_HUMAN	Q00716	26.74	86	56	4	41	123	26	107	0.26	30.4
tr|F8WC99|F8WC99_HUMAN	Q01218	27.5	80	51	4	47	123	30	105	0.53	29.3
tr|F8WC99|F8WC99_HUMAN	Q89182	27.5	80	51	4	47	123	30	105	0.57	29.3

tr|F8WC99|F8WC99_HUMAN	P16561	27.5	80	51	4	47	123	30	105	0.62	29.3
tr|F8WC99|F8WC99_HUMAN	P08714	27.5	80	51	4	47	123	30	105	0.65	28.9
tr|F8WC99|F8WC99_HUMAN	P20978	27.5	80	51	4	47	123	30	105	0.65	28.9
tr|F8WC99|F8WC99_HUMAN	O72737	27.5	80	51	4	47	123	30	105	1	28.5
tr|F8WC99|F8WC99_HUMAN	P12439	35.14	37	23	1	70	106	247	282	3.8	26.9
tr|F8WC99|F8WC99_HUMAN	Q8UYT3	61.54	13	5	0	115	127	67	79	4.7	25.8
tr|F8WC99|F8WC99_HUMAN	P36693	35.29	34	19	1	62	95	260	290	7.7	25.8
tr|F8WC99|F8WC99_HUMAN	P27330	33.33	27	18	0	120	146	5	31	9.7	25.4
tr|F8WEX3|F8WEX3_HUMAN	P0C788	45.33	75	41	0	1	75	63	137	2.00E-18	76.6
tr|F8WEX3|F8WEX3_HUMAN	P27330	34.62	26	17	0	46	71	5	30	1.1	26.2
tr|F8WEX3|F8WEX3_HUMAN	Q04995	32.5	40	27	0	25	64	265	304	1.7	25.8
tr|F8WEX3|F8WEX3_HUMAN	Q05312	32.5	40	27	0	25	64	263	302	1.8	25.8
tr|F8WEX3|F8WEX3_HUMAN	Q8UYT3	61.54	13	5	0	41	53	67	79	3.4	24.6
tr|F8WEX3|F8WEX3_HUMAN	P19030	30	40	28	0	25	64	265	304	4.9	24.6
tr|F8WEX3|F8WEX3_HUMAN	P16090	33.33	39	26	0	26	64	266	304	5.2	24.6

tr|F8WEX3|F8WEX3_HUMAN	P33807	45	20	11	0	30	49	88	107	5.2	24.3
tr|F8WEX3|F8WEX3_HUMAN	Q03804	30	40	28	0	25	64	265	304	5.5	24.3
tr|F8WEX3|F8WEX3_HUMAN	Q02282	32.5	40	27	0	25	64	265	304	7.1	24.3
tr|F8WEX3|F8WEX3_HUMAN	Q04993	30.77	39	27	0	26	64	266	304	7.9	23.9
sp|P41217|OX2G_HUMAN	P0C788	32.71	214	132	2	1	214	1	202	1.00E-34	127
sp|P41217|OX2G_HUMAN	Q89738	24.51	257	142	11	48	258	38	288	5.00E-08	52.4
sp|P41217|OX2G_HUMAN	Q9QJ17	37.29	59	36	1	115	173	120	177	3.00E-06	47
sp|P41217|OX2G_HUMAN	P33807	28.75	80	50	4	47	123	32	107	0.49	30.8
sp|P41217|OX2G_HUMAN	Q00716	26.74	86	56	4	41	123	26	107	0.79	30
sp|P41217|OX2G_HUMAN	Q01218	27.5	80	51	4	47	123	30	105	1.2	29.6
sp|P41217|OX2G_HUMAN	Q89182	27.5	80	51	4	47	123	30	105	1.2	29.6
sp|P41217|OX2G_HUMAN	P08714	27.5	80	51	4	47	123	30	105	1.3	29.6
sp|P41217|OX2G_HUMAN	P16561	27.5	80	51	4	47	123	30	105	1.4	29.3
sp|P41217|OX2G_HUMAN	P20978	27.5	80	51	4	47	123	30	105	1.5	29.3
sp|P41217|OX2G_HUMAN	O72737	27.5	80	51	4	47	123	30	105	2.2	28.9
sp|P41217|OX2G_HUMAN	P0CW72	24.75	101	65	4	113	204	96	194	2.9	28.1
sp|P41217|OX2G_HUMAN	P03228	24.75	101	65	4	113	204	96	194	2.9	28.1
sp|P41217|OX2G_HUMAN	P0C6N0	24.75	101	65	4	113	204	96	194	2.9	28.1
sp|P41217|OX2G_HUMAN	P35934	46.67	30	13	2	166	192	275	304	8	27.3
tr|U3KQG5|U3KQG5_HUMAN	P0C788	36.47	170	108	0	18	187	35	204	2.00E-34	125

tr|U3KQG5|U3KQG5_HUMAN	Q89738	37.29	59	36	1	86	144	119	176	2.00E-07	49.7
tr|U3KQG5|U3KQG5_HUMAN	Q9QJ17	37.29	59	36	1	86	144	120	177	2.00E-06	46.6
tr|U3KQG5|U3KQG5_HUMAN	P33807	28.75	80	50	4	18	94	32	107	0.098	32.3
tr|U3KQG5|U3KQG5_HUMAN	Q01218	27.5	80	51	4	18	94	30	105	0.23	31.2
tr|U3KQG5|U3KQG5_HUMAN	Q89182	27.5	80	51	4	18	94	30	105	0.25	31.2
tr|U3KQG5|U3KQG5_HUMAN	P08714	27.5	80	51	4	18	94	30	105	0.26	30.8
tr|U3KQG5|U3KQG5_HUMAN	P16561	27.5	80	51	4	18	94	30	105	0.27	30.8
tr|U3KQG5|U3KQG5_HUMAN	P20978	27.5	80	51	4	18	94	30	105	0.28	30.8
tr|U3KQG5|U3KQG5_HUMAN	Q00716	26.74	86	56	4	12	94	26	107	0.35	30.4
tr|U3KQG5|U3KQG5_HUMAN	O72737	27.5	80	51	4	18	94	30	105	0.39	30.4
tr|U3KQG5|U3KQG5_HUMAN	P0CW72	26.47	102	62	6	84	175	96	194	2.8	27.7
tr|U3KQG5|U3KQG5_HUMAN	P03228	26.47	102	62	6	84	175	96	194	2.8	27.7
tr|U3KQG5|U3KQG5_HUMAN	P0C6N0	26.47	102	62	6	84	175	96	194	2.8	27.7
tr|U3KQG5|U3KQG5_HUMAN	P35934	46.67	30	13	2	137	163	275	304	4.9	27.3
tr|U3KQG5|U3KQG5_HUMAN	P12439	35.14	37	23	1	41	77	247	282	7.6	26.6

tr|U3KQG5|U3KQG5_HUMAN	Q5GA87	36.21	58	35	2	125	181	173	229	8.1	26.2
tr|U3KQQ2|U3KQQ2_HUMAN	P03253	32.43	37	25	0	2	38	140	176	5.6	23.9
tr|U3KQQ2|U3KQQ2_HUMAN	P03252	32.43	37	25	0	2	38	140	176	5.6	23.9
tr|U3KQQ2|U3KQQ2_HUMAN	P11209	35.48	31	20	0	1	31	1	31	5.6	23.9
tr|U3KQQ2|U3KQQ2_HUMAN	P15630	37.93	29	18	0	20	48	93	121	6.3	23.5
tr|E9PI80|E9PI80_HUMAN	Q00996	50.91	55	26	1	8	62	3	56	1.00E-11	57.4
tr|E9PI80|E9PI80_HUMAN	O71188	31.71	41	28	0	19	59	1573	1613	3.9	26.2
tr|E9PI80|E9PI80_HUMAN	O71189	31.71	41	28	0	19	59	1573	1613	4	26.2
tr|E9PI80|E9PI80_HUMAN	Q86117	33.33	33	22	0	21	53	1124	1156	7	25.4
tr|E9PI80|E9PI80_HUMAN	Q86119	33.33	33	22	0	21	53	1124	1156	7.1	25.4
tr|E9PNW4|E9PNW4_HUMAN	Q00996	51.58	95	45	1	8	102	3	96	2.00E-30	107
tr|E9PNW4|E9PNW4_HUMAN	P69514	33.33	42	25	1	59	97	1173	1214	0.51	28.5
tr|E9PNW4|E9PNW4_HUMAN	Q86117	33.33	33	22	0	21	53	1124	1156	4.2	25.8
tr|E9PNW4|E9PNW4_HUMAN	Q86119	33.33	33	22	0	21	53	1124	1156	4.4	25.8
tr|E9PNW4|E9PNW4_HUMAN	P04883	28.12	32	23	0	56	87	410	441	5.9	25.4
tr|E9PNW4|E9PNW4_HUMAN	P27410	33.33	33	22	0	21	53	1124	1156	6.1	25.4
tr|E9PNW4|E9PNW4_HUMAN	P27411	33.33	33	22	0	21	53	1124	1156	6.2	25.4

tr|E9PNW4|E9PNW4_HUMAN	Q89273	33.33	33	22	0	21	53	1124	1156	6.4	25.4
tr|E9PR17|E9PR17_HUMAN	Q00996	51.58	95	45	1	8	102	3	96	2.00E-30	107
tr|E9PR17|E9PR17_HUMAN	P69514	33.33	42	25	1	59	97	1173	1214	0.86	28.5
tr|E9PR17|E9PR17_HUMAN	Q86117	33.33	33	22	0	21	53	1124	1156	6.1	26.2
tr|E9PR17|E9PR17_HUMAN	Q86119	33.33	33	22	0	21	53	1124	1156	6.1	26.2
tr|E9PR17|E9PR17_HUMAN	P04883	28.12	32	23	0	56	87	410	441	7.9	25.4
tr|E9PR17|E9PR17_HUMAN	P12911	35.48	31	20	0	99	129	110	140	8.3	25.4
tr|E9PR17|E9PR17_HUMAN	P27410	33.33	33	22	0	21	53	1124	1156	8.8	25.4
tr|E9PR17|E9PR17_HUMAN	Q89273	33.33	33	22	0	21	53	1124	1156	8.8	25.4
tr|E9PR17|E9PR17_HUMAN	P27411	33.33	33	22	0	21	53	1124	1156	8.8	25.4
tr|H0YET2|H0YET2_HUMAN	Q00996	48.48	33	17	0	2	34	72	104	7.00E-06	39.3
tr|H0YET2|H0YET2_HUMAN	Q775J3	48.39	31	16	0	17	47	513	543	0.077	29.3
tr|H0YET2|H0YET2_HUMAN	P09260	48.39	31	16	0	17	47	513	543	0.077	29.3
tr|H0YET2|H0YET2_HUMAN	P10190	33.33	45	28	1	2	46	530	572	1	25.8
tr|H0YET2|H0YET2_HUMAN	P18557	33.33	48	28	2	5	48	32	79	2.1	23.9

tr|H0YET2|H0YET2_HUMAN	P0C9H4	33.33	48	28	2	5	48	32	79	2.4	24.3
tr|H0YET2|H0YET2_HUMAN	P0C9H6	33.33	48	28	2	5	48	32	79	2.4	24.3
tr|H0YET2|H0YET2_HUMAN	P26705	33.33	48	28	2	5	48	32	79	2.9	23.9
tr|H0YET2|H0YET2_HUMAN	Q9E6Q9	36.36	33	21	0	17	49	148	180	4.6	23.5
tr|H0YET2|H0YET2_HUMAN	P12726	33.33	33	21	1	21	52	370	402	4.9	23.5
tr|H0YET2|H0YET2_HUMAN	P04048	61.54	13	5	0	8	20	107	119	7.3	23.1
tr|H0YET2|H0YET2_HUMAN	P0C9H5	31.25	48	29	2	5	48	32	79	7.7	22.7
tr|H0YET2|H0YET2_HUMAN	P26709	43.48	23	13	0	5	27	38	60	9.1	22.7
tr|H0YET2|H0YET2_HUMAN	Q6DN67	42.86	21	12	0	7	27	578	598	9.9	22.7
sp|P13987|CD59_HUMAN	Q00996	49.51	103	51	1	8	110	3	104	7.00E-32	111
sp|P13987|CD59_HUMAN	Q775J3	48.39	31	16	0	93	123	513	543	0.47	29.3
sp|P13987|CD59_HUMAN	P09260	48.39	31	16	0	93	123	513	543	0.47	29.3
sp|P13987|CD59_HUMAN	P69514	33.33	42	25	1	59	97	1173	1214	0.82	28.5
sp|P13987|CD59_HUMAN	P0C6F3	28.38	74	45	2	29	102	3930	3995	1.2	28.1
sp|P13987|CD59_HUMAN	P0C6V7	28.38	74	45	2	29	102	3930	3995	1.3	28.1
sp|P13987|CD59_HUMAN	P10190	32.61	46	29	1	77	122	529	572	4.7	26.2
sp|P13987|CD59_HUMAN	Q86117	33.33	33	22	0	21	53	1124	1156	6.6	25.8
sp|P13987|CD59_HUMAN	Q86119	33.33	33	22	0	21	53	1124	1156	6.8	25.8
sp|P13987|CD59_HUMAN	P04883	28.12	32	23	0	56	87	410	441	9.1	25.4
sp|P13987|CD59_HUMAN	P27411	33.33	33	22	0	21	53	1124	1156	9.3	25.4

sp|P13987|CD59_HUMAN	P27410	33.33	33	22	0	21	53	1124	1156	9.4	25.4
sp|P13987|CD59_HUMAN	Q89273	33.33	33	22	0	21	53	1124	1156	9.5	25.4
tr|B4E009|B4E009_HUMAN	P14371	33.71	89	58	1	76	163	39	127	9.00E-14	65.1
tr|B4E009|B4E009_HUMAN	P14370	30.09	113	68	5	41	150	12	116	2.00E-10	55.5
tr|B4E009|B4E009_HUMAN	O89335	35	60	38	1	85	144	66	124	2.00E-07	47.4
tr|B4E009|B4E009_HUMAN	Q65150	36.07	61	36	2	85	143	67	126	2.00E-06	44.3
tr|B4E009|B4E009_HUMAN	P24765	32.26	62	38	2	85	144	56	115	4.00E-06	43.5
tr|B4E009|B4E009_HUMAN	P0CA63	33.33	75	46	3	49	121	26	98	4.00E-06	43.5
tr|B4E009|B4E009_HUMAN	P21063	28.99	69	44	3	79	144	49	115	8.00E-06	43.1
tr|B4E009|B4E009_HUMAN	P0CA65	31.34	67	39	3	85	144	63	129	2.00E-04	38.9
tr|B4E009|B4E009_HUMAN	P14372	26.98	63	33	1	52	114	16	65	0.001	36.6
tr|B4E009|B4E009_HUMAN	P0CA64	26.15	65	43	1	85	144	66	130	0.002	36.2
tr|B4E009|B4E009_HUMAN	Q9ICC1	25.84	89	55	4	84	161	20	108	0.004	35
tr|B4E009|B4E009_HUMAN	P21057	25	80	58	1	83	162	45	122	0.01	34.3
tr|B4E009|B4E009_HUMAN	P24761	25	80	58	1	83	162	45	122	0.014	33.9
tr|B4E009|B4E009_HUMAN	P33851	23.75	80	59	1	83	162	45	122	0.039	32.7

tr|B4E009|B4E009_HUMAN	Q66653	25	60	44	1	95	153	114	173	0.058	32.3
tr|B4E009|B4E009_HUMAN	Q1HVG2	21.7	106	64	4	69	161	83	182	0.14	31.2
tr|B4E009|B4E009_HUMAN	Q83017	27.78	90	55	3	49	136	1835	1916	0.23	30.8
tr|B4E009|B4E009_HUMAN	P07065	25.4	63	40	2	64	122	213	272	1.1	28.9
tr|B4E009|B4E009_HUMAN	P0C6Z5	21.7	106	64	4	69	161	83	182	1.2	28.5
tr|B4E009|B4E009_HUMAN	P03205	21.7	106	64	4	69	161	83	182	1.2	28.5
tr|B4E009|B4E009_HUMAN	Q65652	33.33	45	26	2	112	152	946	990	7.3	26.2
sp|Q07108|CD69_HUMAN	P14371	31.4	121	82	1	76	195	39	159	5.00E-18	77.8
sp|Q07108|CD69_HUMAN	P14370	26.03	146	95	5	41	186	12	144	3.00E-12	61.6
sp|Q07108|CD69_HUMAN	O89335	35	60	38	1	85	144	66	124	4.00E-07	47
sp|Q07108|CD69_HUMAN	Q65150	36.07	61	36	2	85	143	67	126	5.00E-06	44.3
sp|Q07108|CD69_HUMAN	P24765	30.43	69	43	3	79	144	49	115	7.00E-06	43.9
sp|Q07108|CD69_HUMAN	P0CA63	33.33	75	46	3	49	121	26	98	9.00E-06	43.1
sp|Q07108|CD69_HUMAN	P21063	28.99	69	44	3	79	144	49	115	2.00E-05	42.7
sp|Q07108|CD69_HUMAN	P0CA65	31.34	67	39	3	85	144	63	129	5.00E-04	38.5

sp|Q07108|CD69_HUMAN	P14372	26.98	63	33	1	52	114	16	65	0.002	36.6
sp|Q07108|CD69_HUMAN	P21057	21.77	124	87	2	83	198	45	166	0.002	36.6
sp|Q07108|CD69_HUMAN	P24761	21.77	124	87	2	83	198	45	166	0.003	36.6
sp|Q07108|CD69_HUMAN	P0CA64	26.15	65	43	1	85	144	66	130	0.004	35.8
sp|Q07108|CD69_HUMAN	Q9ICC1	25.84	89	55	4	84	161	20	108	0.007	34.7
sp|Q07108|CD69_HUMAN	P33851	20.97	124	88	2	83	198	45	166	0.01	34.7
sp|Q07108|CD69_HUMAN	Q66653	25	60	44	1	95	153	114	173	0.1	32
sp|Q07108|CD69_HUMAN	Q1HVG2	22.39	134	80	7	69	186	83	208	0.2	31.2
sp|Q07108|CD69_HUMAN	Q83017	27.78	90	55	3	49	136	1835	1916	0.27	31.2
sp|Q07108|CD69_HUMAN	P0C6Z5	22.39	134	80	7	69	186	83	208	1.5	28.5
sp|Q07108|CD69_HUMAN	P03205	22.39	134	80	7	69	186	83	208	1.5	28.5
sp|Q07108|CD69_HUMAN	P07065	25.86	58	36	2	64	117	213	267	1.6	28.5
sp|Q07108|CD69_HUMAN	P22653	19.61	153	90	5	46	197	53	173	2.1	28.1
sp|Q07108|CD69_HUMAN	P25328	41.67	24	11	1	142	165	654	674	2.3	28.1
sp|Q07108|CD69_HUMAN	P22652	19.61	153	90	5	46	197	53	173	2.8	27.7
sp|Q07108|CD69_HUMAN	P16804	26.92	78	56	1	119	195	2	79	6.7	25.8
sp|P46094|XCR1_HUMAN	Q89609	38.81	268	144	6	49	300	90	353	5.00E-45	159
sp|P46094|XCR1_HUMAN	F5HF62	30.92	304	191	6	13	305	14	309	1.00E-37	139
sp|P46094|XCR1_HUMAN	P69333	30.92	304	191	6	13	305	14	309	2.00E-37	138
sp|P46094|XCR1_HUMAN	P69332	30.92	304	191	6	13	305	14	309	2.00E-37	138
sp|P46094|XCR1_HUMAN	Q86917	32.35	272	172	6	37	298	97	366	2.00E-37	139
sp|P46094|XCR1_HUMAN	Q08520	27.3	282	188	4	36	301	64	344	8.00E-33	125

sp|P46094|XCR1_HUMAN	P32229	25.51	243	165	3	75	301	1	243	2.00E-25	103
sp|P46094|XCR1_HUMAN	Q9J5H4	23.84	281	186	11	29	290	25	296	5.00E-16	77
sp|P46094|XCR1_HUMAN	P09703	22.44	303	217	7	31	315	20	322	4.00E-12	65.9
sp|P46094|XCR1_HUMAN	Q9J529	24.64	280	188	9	31	291	17	292	6.00E-12	64.7
sp|P46094|XCR1_HUMAN	F5HDK1	22.44	303	217	7	31	315	22	324	2.00E-11	63.5
sp|P46094|XCR1_HUMAN	Q9J5I0	31.3	115	76	2	16	128	2	115	2.00E-10	60.5
sp|P46094|XCR1_HUMAN	Q98146	23.31	296	213	5	15	298	32	325	3.00E-08	53.9
sp|P46094|XCR1_HUMAN	Q01035	23.72	274	198	5	33	298	41	311	7.00E-07	49.3
sp|P46094|XCR1_HUMAN	Q83207	18.56	291	206	4	63	328	67	351	4.00E-04	41.2
sp|P46094|XCR1_HUMAN	P16849	20.69	261	182	4	64	303	66	322	0.007	37.4
sp|P46094|XCR1_HUMAN	P52381	20.5	278	181	7	58	295	42	319	0.047	34.3
sp|P46094|XCR1_HUMAN	Q18LE5	22.22	90	66	2	43	128	32	121	0.091	33.5
sp|P46094|XCR1_HUMAN	Q6SW98	21.51	93	73	0	211	303	230	322	0.1	33.5
sp|P46094|XCR1_HUMAN	O12000	21.3	108	85	0	205	312	223	330	0.11	33.1
sp|P46094|XCR1_HUMAN	P52380	25	104	78	0	203	306	210	313	0.29	32
sp|P46094|XCR1_HUMAN	Q66673	22.77	101	77	1	205	304	230	330	0.31	32
sp|P46094|XCR1_HUMAN	P19560	47.37	19	10	0	310	328	377	395	4.9	28.5
tr|Q689E2|Q689E2_HUMAN	Q6R7D6	34.62	26	16	1	5	29	165	190	6.6	22.7
sp|P32246|CCR1_HUMAN	Q89609	51.43	315	152	1	21	334	59	373	7.00E	306

										-101	
sp|P32246|CCR1_HUMAN	Q86917	35.99	289	179	4	27	315	87	369	4.00E-60	200
sp|P32246|CCR1_HUMAN	Q08520	32.98	282	175	7	43	315	68	344	8.00E-49	170
sp|P32246|CCR1_HUMAN	F5HF62	30.9	343	220	8	15	352	13	343	2.00E-47	166
sp|P32246|CCR1_HUMAN	P69333	31.13	318	204	7	15	327	13	320	7.00E-46	162
sp|P32246|CCR1_HUMAN	P69332	31.13	318	204	7	15	327	13	320	7.00E-46	162
sp|P32246|CCR1_HUMAN	P32229	31.4	242	153	6	78	310	1	238	2.00E-38	140
sp|P32246|CCR1_HUMAN	P09703	25.26	293	214	2	43	334	39	327	3.00E-28	113
sp|P32246|CCR1_HUMAN	F5HDK1	25.6	293	213	2	43	334	41	329	3.00E-28	113
sp|P32246|CCR1_HUMAN	Q9J5H4	24.08	299	198	10	25	304	8	296	2.00E-23	98.2
sp|P32246|CCR1_HUMAN	Q9J5I0	31.86	295	181	9	17	306	4	283	1.00E-21	93.2
sp|P32246|CCR1_HUMAN	P16849	25.38	331	206	11	43	354	41	349	1.00E-21	94.4
sp|P32246|CCR1_HUMAN	Q6SW98	24.85	330	208	11	43	354	42	349	2.00E-20	90.9
sp|P32246|CCR1_HUMAN	Q9J529	24.3	284	187	9	43	310	26	297	6.00E-16	76.6
sp|P32246|CCR1_HUMAN	Q83207	22.43	321	216	9	42	346	41	344	2.00E-14	73.2
sp|P32246|CCR1_HUMAN	O12000	23.08	299	203	10	45	323	41	332	7.00E	68.6

										-13	
sp|P32246|CCR1_HUMAN	Q66673	22.88	319	236	6	2	318	20	330	1.00E-11	64.7
sp|P32246|CCR1_HUMAN	Q98146	21.3	277	216	2	42	317	55	330	9.00E-10	58.5
sp|P32246|CCR1_HUMAN	P52380	22.96	270	197	7	53	317	47	310	1.00E-09	58.2
sp|P32246|CCR1_HUMAN	Q01035	20.58	311	221	8	9	310	16	309	7.00E-09	55.8
sp|P32246|CCR1_HUMAN	Q18LE5	23.81	231	164	7	40	268	29	249	7.00E-06	46.6
sp|P32246|CCR1_HUMAN	P52381	20.38	314	221	10	8	309	23	319	1.00E-04	42.4
sp|P32246|CCR1_HUMAN	P42540	40.74	27	16	0	316	342	25	51	2.7	28.9
tr|E9PH76|E9PH76_HUMAN	Q89609	53.76	93	41	1	29	119	59	151	2.00E-15	71.2
tr|E9PH76|E9PH76_HUMAN	F5HF62	37.37	99	61	1	24	121	14	112	7.00E-15	69.3
tr|E9PH76|E9PH76_HUMAN	P69333	37.37	99	61	1	24	121	14	112	1.00E-14	68.6
tr|E9PH76|E9PH76_HUMAN	P69332	37.37	99	61	1	24	121	14	112	1.00E-14	68.6
tr|E9PH76|E9PH76_HUMAN	Q08520	40.54	74	43	1	48	121	65	137	9.00E-14	66.2
tr|E9PH76|E9PH76_HUMAN	Q86917	37.35	83	45	3	45	121	94	175	7.00E-10	55.1
tr|E9PH76|E9PH76_HUMAN	Q9J5I0	35.48	93	55	3	25	115	2	91	2.00E-06	44.3
tr|E9PH76|E9PH76_HUMAN	Q9J5H4	30.59	85	53	4	33	115	21	101	3.00E-05	41.2

tr|E9PH76|E9PH76_HUMAN	P32229	36.11	36	23	0	86	121	1	36	6.00E-05	40
tr|E9PH76|E9PH76_HUMAN	F5HDK1	39.29	56	33	1	51	105	41	96	0.006	34.3
tr|E9PH76|E9PH76_HUMAN	Q18LE5	26.14	88	63	2	35	121	16	102	0.006	34.3
tr|E9PH76|E9PH76_HUMAN	P09703	32.76	58	35	1	52	105	37	94	0.023	32.7
tr|E9PH76|E9PH76_HUMAN	Q9J529	36.36	44	26	1	78	119	52	95	0.092	30.8
tr|E9PH76|E9PH76_HUMAN	Q999T0	36.59	41	24	1	31	69	729	769	0.23	30
tr|E9PH76|E9PH76_HUMAN	P05842	33.33	45	23	1	71	115	192	229	2.4	26.9
tr|E9PH76|E9PH76_HUMAN	Q8B912	32.26	62	32	2	47	98	1647	1708	3.5	26.6
tr|E9PH76|E9PH76_HUMAN	Q9YN02	32.26	62	32	2	47	98	1647	1708	3.9	26.6
tr|E9PH76|E9PH76_HUMAN	Q9WJB2	32.26	62	32	2	47	98	1647	1708	4.5	26.2
tr|E9PH76|E9PH76_HUMAN	Q98146	25.56	90	60	2	12	95	12	100	5	25.8
tr|E9PH76|E9PH76_HUMAN	Q6UDM4	48	25	12	1	27	50	66	90	9.9	25
sp|P41597|CCR2_HUMAN	Q89609	50.68	296	138	4	29	317	59	353	1.00E-82	260
sp|P41597|CCR2_HUMAN	Q86917	33.46	272	174	4	45	312	94	362	2.00E-48	170
sp|P41597|CCR2_HUMAN	F5HF62	31	300	199	5	24	321	14	307	4.00E-46	163

sp|P41597|CCR2_HUMAN	P69333	31	300	199	5	24	321	14	307	6.00E-46	162
sp|P41597|CCR2_HUMAN	P69332	31	300	199	5	24	321	14	307	6.00E-46	162
sp|P41597|CCR2_HUMAN	Q08520	32.48	274	173	5	51	314	68	339	1.00E-43	156
sp|P41597|CCR2_HUMAN	P32229	30.13	239	156	4	86	314	1	238	5.00E-35	130
sp|P41597|CCR2_HUMAN	F5HDK1	26.57	271	186	6	51	314	41	305	3.00E-17	81.3
sp|P41597|CCR2_HUMAN	P09703	25.38	264	185	5	57	314	46	303	1.00E-16	79.7
sp|P41597|CCR2_HUMAN	P16849	22.81	342	225	11	51	370	41	365	2.00E-15	76.6
sp|P41597|CCR2_HUMAN	Q9J5H4	22.84	289	197	9	33	308	21	296	2.00E-15	75.9
sp|P41597|CCR2_HUMAN	Q9J529	24.59	244	171	7	78	310	52	293	3.00E-15	74.7
sp|P41597|CCR2_HUMAN	Q6SW98	22.58	341	226	11	51	370	42	365	4.00E-15	75.5
sp|P41597|CCR2_HUMAN	O12000	20.97	310	212	10	79	362	72	374	4.00E-13	69.3
sp|P41597|CCR2_HUMAN	Q83207	21.17	274	197	7	79	338	72	340	1.00E-12	67.8
sp|P41597|CCR2_HUMAN	Q9J5I0	26.78	295	194	8	25	310	2	283	2.00E-12	67
sp|P41597|CCR2_HUMAN	Q01035	23.64	330	221	11	16	332	8	319	2.00E-09	57.8
sp|P41597|CCR2_HUMAN	Q98146	21.59	315	230	7	12	313	12	322	7.00E-07	49.7

sp|P41597|CCR2_HUMAN	P52381	19.47	262	187	4	76	324	80	330	4.00E-05	44.3
sp|P41597|CCR2_HUMAN	Q18LE5	24.64	138	98	3	35	167	16	152	2.00E-04	42.4
sp|P41597|CCR2_HUMAN	P52380	18.67	300	221	7	77	372	65	345	8.00E-04	40
sp|P41597|CCR2_HUMAN	A4ZUD1	38.33	60	33	2	120	179	162	217	2	29.6
sp|P41597|CCR2_HUMAN	Q999T0	36.59	41	24	1	31	69	729	769	2.2	29.6
sp|P41597|CCR2_HUMAN	P16838	43.9	41	16	2	228	268	69	102	3.2	27.7
sp|P51677|CCR3_HUMAN	Q89609	55.13	312	139	1	24	334	62	373	2.00E-109	328
sp|P51677|CCR3_HUMAN	Q86917	34.8	273	175	3	40	312	97	366	1.00E-54	186
sp|P51677|CCR3_HUMAN	Q08520	31.43	280	172	8	43	310	68	339	7.00E-43	154
sp|P51677|CCR3_HUMAN	F5HF62	28.83	333	224	6	17	348	16	336	5.00E-42	151
sp|P51677|CCR3_HUMAN	P69333	29.97	297	197	5	17	312	16	302	1.00E-41	150
sp|P51677|CCR3_HUMAN	P69332	29.97	297	197	5	17	312	16	302	1.00E-41	150
sp|P51677|CCR3_HUMAN	P32229	30.33	244	151	7	78	309	1	237	3.00E-34	128
sp|P51677|CCR3_HUMAN	P09703	28.41	271	185	5	43	310	39	303	4.00E-26	106
sp|P51677|CCR3_HUMAN	F5HDK1	27.41	270	189	4	43	310	41	305	5.00E-26	106
sp|P51677|CCR3_HUMAN	Q9J5I0	31.28	227	137	7	40	257	23	239	5.00E-20	88.6

sp|P51677|CCR3_HUMAN	Q9J529	27.24	279	185	10	43	310	26	297	2.00E-19	86.7
sp|P51677|CCR3_HUMAN	Q6SW98	23.38	325	215	9	43	352	42	347	8.00E-19	86.3
sp|P51677|CCR3_HUMAN	P16849	23.31	326	215	9	43	352	41	347	2.00E-18	85.1
sp|P51677|CCR3_HUMAN	Q9J5H4	24.73	279	181	10	40	304	33	296	1.00E-17	82
sp|P51677|CCR3_HUMAN	Q83207	24.02	333	221	10	39	352	49	368	2.00E-15	76.3
sp|P51677|CCR3_HUMAN	O12000	22.18	275	204	5	43	310	43	314	8.00E-14	71.2
sp|P51677|CCR3_HUMAN	P52380	22.04	304	226	7	53	349	47	346	1.00E-08	55.1
sp|P51677|CCR3_HUMAN	Q01035	21.58	278	198	7	40	310	45	309	1.00E-07	52
sp|P51677|CCR3_HUMAN	Q98146	23.44	337	225	11	1	317	7	330	3.00E-07	50.4
sp|P51677|CCR3_HUMAN	Q18LE5	21.69	249	177	7	28	271	17	252	2.00E-05	45.4
sp|P51677|CCR3_HUMAN	P27964	25	64	39	2	48	106	265	324	0.19	32.7
sp|P51677|CCR3_HUMAN	P50793	28.12	64	37	3	48	106	263	322	0.25	32.3
sp|P51677|CCR3_HUMAN	P54669	25	64	39	2	48	106	265	324	0.3	32.3
sp|P51677|CCR3_HUMAN	P52381	20.79	101	80	0	209	309	219	319	0.46	31.6
sp|P51677|CCR3_HUMAN	P24838	23.44	64	40	3	48	106	264	323	0.78	30.8
sp|P51677|CCR3_HUMAN	Q02274	26.15	65	39	3	47	106	261	321	0.78	30.8
sp|P51677|CCR3_HUMAN	Q5UR54	25.86	58	43	0	276	333	116	173	3.3	28.1
sp|P51677|CCR3_HUMAN	Q2YHF0	28.89	45	27	1	90	129	230	274	4.5	28.5

sp|P51677|CCR3_HUMAN	P0C6V6	26.27	118	72	5	193	310	3423	3525	5.7	28.5
sp|P51677|CCR3_HUMAN	P0C6Y4	26.27	118	72	5	193	310	3423	3525	5.8	28.5
sp|P51677|CCR3_HUMAN	Q5UCB8	26.67	45	28	1	90	129	230	274	7	28.1
sp|P51677|CCR3_HUMAN	Q58HT7	26.67	45	28	1	90	129	230	274	7	28.1
sp|P51677|CCR3_HUMAN	Q2YHF2	26.67	45	28	1	90	129	230	274	7	28.1
sp|P51677|CCR3_HUMAN	Q88668	26.67	45	28	1	90	129	230	274	7	28.1
sp|P51677|CCR3_HUMAN	Q8V5U0	32.31	65	40	3	39	99	275	339	9.7	27.3
sp|P51677|CCR3_HUMAN	P36797	25.49	51	36	2	234	284	320	368	9.7	27.3
tr|Q8TDP5|Q8TDP5_HUMAN	Q86917	43.9	41	22	1	40	80	97	136	5.00E-05	39.3
tr|Q8TDP5|Q8TDP5_HUMAN	Q89609	59.65	57	23	0	24	80	62	118	6.00E-04	36.2
tr|Q8TDP5|Q8TDP5_HUMAN	F5HF62	36.59	41	26	0	40	80	39	79	0.001	35
tr|Q8TDP5|Q8TDP5_HUMAN	P69333	36.59	41	26	0	40	80	39	79	0.002	35
tr|Q8TDP5|Q8TDP5_HUMAN	P69332	36.59	41	26	0	40	80	39	79	0.002	35
tr|Q8TDP5|Q8TDP5_HUMAN	Q08520	41.46	41	23	1	40	80	65	104	0.003	34.3
tr|Q8TDP5|Q8TDP5_HUMAN	Q9J5I0	41.46	41	23	1	40	80	23	62	0.014	32
tr|Q8TDP5|Q8TDP5_HUMAN	Q9J5H4	31.71	41	27	1	40	80	33	72	0.021	31.6
tr|Q8TDP5|Q8TDP5_HUMAN	P09703	35.9	39	24	1	43	80	39	77	2.1	25.8
tr|Q8TDP5|Q8TDP5_HUM	Q98146	33.33	39	26	0	42	80	55	93	4.8	24.6

AN											
tr|Q8TDP5|Q8TDP5_HUMAN	Q8V5U0	40	40	23	1	40	78	276	315	5.5	24.6
tr|Q8TDP5|Q8TDP5_HUMAN	P22958	42.86	21	12	0	18	38	526	546	7.2	24.3
sp|P51679|CCR4_HUMAN	Q89609	40.71	312	181	3	28	336	61	371	4.00E-66	216
sp|P51679|CCR4_HUMAN	Q86917	40	300	172	5	28	324	81	375	7.00E-66	215
sp|P51679|CCR4_HUMAN	Q08520	37.77	278	165	4	45	315	65	341	4.00E-53	182
sp|P51679|CCR4_HUMAN	F5HF62	33.11	293	187	3	22	313	16	300	7.00E-51	175
sp|P51679|CCR4_HUMAN	P69333	33.11	293	187	3	22	313	16	300	9.00E-51	175
sp|P51679|CCR4_HUMAN	P69332	33.11	293	187	3	22	313	16	300	9.00E-51	175
sp|P51679|CCR4_HUMAN	P32229	37.5	240	143	3	83	315	1	240	3.00E-43	152
sp|P51679|CCR4_HUMAN	P09703	28.11	281	192	6	48	322	39	315	8.00E-28	111
sp|P51679|CCR4_HUMAN	F5HDK1	28.11	281	192	6	48	322	41	317	1.00E-27	111
sp|P51679|CCR4_HUMAN	Q9J5H4	24.37	279	196	7	37	307	25	296	3.00E-25	103
sp|P51679|CCR4_HUMAN	Q9J5I0	26.35	296	198	10	30	317	10	293	4.00E-19	85.9
sp|P51679|CCR4_HUMAN	Q9J529	25.09	275	186	8	48	309	26	293	3.00E-14	71.6
sp|P51679|CCR4_HUMAN	Q6SW9	22.95	292	195	10	48	320	42	322	6.00E	71.6

	8									-14	
sp|P51679|CCR4_HUMAN	P16849	22.53	293	196	9	48	320	41	322	6.00E-13	68.9
sp|P51679|CCR4_HUMAN	Q98146	24.38	283	212	2	47	327	55	337	2.00E-12	66.6
sp|P51679|CCR4_HUMAN	Q01035	24.92	321	216	8	12	321	11	317	8.00E-12	64.7
sp|P51679|CCR4_HUMAN	Q83207	24.55	330	212	10	8	317	8	320	1.00E-11	64.7
sp|P51679|CCR4_HUMAN	O12000	24.04	312	201	12	72	360	68	366	9.00E-10	58.9
sp|P51679|CCR4_HUMAN	Q66673	21.09	294	213	8	36	320	46	329	0.001	40
sp|P51679|CCR4_HUMAN	P52380	20.74	270	201	8	59	321	48	311	0.33	32
sp|P51679|CCR4_HUMAN	P52381	25.24	103	73	2	212	312	219	319	0.44	31.6
sp|P51679|CCR4_HUMAN	Q18LE5	25.86	58	42	1	80	136	64	121	0.97	30.4
sp|P51679|CCR4_HUMAN	Q1A243	31.58	38	26	0	21	58	277	314	3.2	28.9
sp|P51679|CCR4_HUMAN	Q88899	30.16	63	42	1	48	110	166	226	5.9	27.7
sp|P51681|CCR5_HUMAN	Q89609	47.69	325	162	3	17	334	59	382	1.00E-89	276
sp|P51681|CCR5_HUMAN	Q86917	35.36	280	172	6	33	309	94	367	5.00E-51	176
sp|P51681|CCR5_HUMAN	Q08520	34.16	281	169	7	36	306	65	339	2.00E-48	169
sp|P51681|CCR5_HUMAN	F5HF62	27.46	335	230	6	17	349	20	343	2.00E-44	158
sp|P51681|CCR5_HUMAN	P69333	28.91	294	196	6	17	308	20	302	2.00E-42	152
sp|P51681|CCR5_HUMAN	P69332	28.91	294	196	6	17	308	20	302	2.00E-42	152
sp|P51681|CCR5_HUMAN	P32229	32.1	243	150	6	74	306	1	238	1.00E	140

										-38	
sp|P51681|CCR5_HUMAN	P09703	26.26	297	203	7	39	332	39	322	2.00E-23	98.6
sp|P51681|CCR5_HUMAN	F5HDK1	25.93	297	204	7	39	332	41	324	2.00E-23	98.2
sp|P51681|CCR5_HUMAN	Q9J5H4	22.38	277	196	7	33	300	30	296	7.00E-16	76.6
sp|P51681|CCR5_HUMAN	Q9J529	23.67	245	176	6	66	302	52	293	8.00E-16	76.3
sp|P51681|CCR5_HUMAN	Q6SW98	20.17	362	262	10	2	351	10	356	3.00E-14	72.8
sp|P51681|CCR5_HUMAN	Q83207	20.38	319	235	9	38	345	41	351	1.00E-13	70.9
sp|P51681|CCR5_HUMAN	P16849	20.55	326	236	9	39	351	41	356	7.00E-13	68.6
sp|P51681|CCR5_HUMAN	Q9J5I0	26.64	304	201	9	11	307	2	290	9.00E-13	67.4
sp|P51681|CCR5_HUMAN	O12000	21.23	292	214	10	39	319	42	328	4.00E-12	66.2
sp|P51681|CCR5_HUMAN	P52380	22.38	286	213	6	65	347	65	344	5.00E-09	56.2
sp|P51681|CCR5_HUMAN	Q01035	22.02	327	214	12	11	323	22	321	8.00E-06	46.2
sp|P51681|CCR5_HUMAN	Q98146	22.65	287	202	8	38	314	55	331	1.00E-05	45.8
sp|P51681|CCR5_HUMAN	P52381	20.65	247	188	5	62	305	78	319	2.00E-05	45.1
sp|P51681|CCR5_HUMAN	Q66673	19.75	314	241	6	2	314	27	330	0.009	37
sp|P51681|CCR5_HUMAN	Q18LE5	21.17	137	102	3	24	155	17	152	0.056	34.3
sp|P51681|CCR5_HUMAN	P0C6Z4	26.67	90	61	2	138	222	11	100	5.8	26.6

sp|P51681|CCR5_HUMAN	P03196	26.67	90	61	2	138	222	11	100	5.8	26.6
sp|P51681|CCR5_HUMAN	P0C6Z3	26.67	90	61	2	138	222	11	100	5.8	26.6
sp|P51681|CCR5_HUMAN	P16838	48.28	29	14	1	220	248	69	96	6.1	26.9
sp|P51681|CCR5_HUMAN	A4ZUD1	35	60	35	2	108	167	162	217	6.9	27.7
sp|P51684|CCR6_HUMAN	F5HF62	30.51	354	215	9	27	373	14	343	3.00E-44	158
sp|P51684|CCR6_HUMAN	Q89609	32.96	267	171	4	64	327	90	351	7.00E-44	157
sp|P51684|CCR6_HUMAN	P69333	31.9	326	193	8	27	347	14	315	3.00E-43	155
sp|P51684|CCR6_HUMAN	P69332	31.9	326	193	8	27	347	14	315	3.00E-43	155
sp|P51684|CCR6_HUMAN	Q08520	31.64	275	175	8	58	325	71	339	9.00E-38	140
sp|P51684|CCR6_HUMAN	Q86917	29.39	279	189	6	53	330	98	369	8.00E-35	132
sp|P51684|CCR6_HUMAN	P32229	29.63	243	159	7	90	325	1	238	2.00E-28	112
sp|P51684|CCR6_HUMAN	F5HDK1	25.61	289	194	8	55	339	41	312	3.00E-21	92.8
sp|P51684|CCR6_HUMAN	P09703	25.91	301	198	9	43	339	31	310	6.00E-21	92
sp|P51684|CCR6_HUMAN	Q9J5H4	22.73	286	205	9	40	319	21	296	2.00E-17	81.6
sp|P51684|CCR6_HUMAN	Q6SW98	23.8	374	234	13	23	371	7	354	9.00E-17	80.5
sp|P51684|CCR6_HUMAN	P16849	24.41	340	208	13	55	371	41	354	4.00E-15	75.5
sp|P51684|CCR6_HUMAN	Q9J529	21.72	290	206	8	44	321	13	293	4.00E	74.3

										-15	
sp|P51684|CCR6_HUMAN	Q83207	25.09	271	187	7	79	340	68	331	2.00E-13	70.1
sp|P51684|CCR6_HUMAN	O12000	25.17	302	203	10	55	343	42	333	3.00E-12	67
sp|P51684|CCR6_HUMAN	P52380	23	313	217	9	65	367	47	345	8.00E-09	55.8
sp|P51684|CCR6_HUMAN	Q9J5I0	24.03	283	186	11	58	329	29	293	2.00E-08	54.7
sp|P51684|CCR6_HUMAN	Q98146	23.3	352	232	11	1	334	1	332	3.00E-08	54.3
sp|P51684|CCR6_HUMAN	P52381	23	313	224	7	36	340	35	338	5.00E-08	53.5
sp|P51684|CCR6_HUMAN	Q01035	27.36	106	76	1	221	325	204	309	5.00E-06	47
sp|P51684|CCR6_HUMAN	Q18LE5	20.16	129	102	1	43	170	20	148	0.007	37.4
sp|P51684|CCR6_HUMAN	Q8JVC1	46.15	26	14	0	30	55	330	355	3.5	28.9
sp|P51684|CCR6_HUMAN	P0C9B5	26.73	101	61	2	116	215	2	90	7.7	27.3
sp|P51684|CCR6_HUMAN	Q65181	26.73	101	61	2	116	215	2	90	7.7	27.3
sp|P51684|CCR6_HUMAN	Q65224	26.73	101	61	2	116	215	2	90	8.6	27.3
sp|P51684|CCR6_HUMAN	P0C9B4	26.73	101	61	2	116	215	2	90	8.7	27.3
tr|J3KSS9|J3KSS9_HUMAN	Q89609	36.58	298	183	4	42	336	62	356	1.00E-46	165
tr|J3KSS9|J3KSS9_HUMAN	Q08520	33.33	282	177	7	57	331	64	341	6.00E-45	160
tr|J3KSS9|J3KSS9_HUMAN	F5HF62	30.21	341	223	4	34	371	15	343	2.00E-41	150
tr|J3KSS9|J3KSS9_HUMAN	P69333	31.05	306	197	3	34	337	15	308	2.00E-40	147
tr|J3KSS9|J3KSS9_HUMAN	P69332	31.05	306	197	3	34	337	15	308	2.00E	147

										-40	
tr|J3KSS9|J3KSS9_HUMAN	P32229	31.69	243	156	6	96	331	1	240	5.00E-36	133
tr|J3KSS9|J3KSS9_HUMAN	Q86917	31.21	282	180	9	58	334	97	369	9.00E-32	123
tr|J3KSS9|J3KSS9_HUMAN	F5HDK1	29.32	324	195	12	30	345	24	321	1.00E-23	99.8
tr|J3KSS9|J3KSS9_HUMAN	P09703	28.47	295	187	10	61	345	39	319	2.00E-21	93.2
tr|J3KSS9|J3KSS9_HUMAN	Q9J5H4	25.18	278	188	10	55	323	30	296	4.00E-17	80.9
tr|J3KSS9|J3KSS9_HUMAN	Q9J529	23.27	245	172	7	90	324	54	292	2.00E-15	75.5
tr|J3KSS9|J3KSS9_HUMAN	P52381	22.73	264	192	6	78	336	71	327	2.00E-10	61.2
tr|J3KSS9|J3KSS9_HUMAN	P52380	21.12	303	222	7	72	365	48	342	2.00E-10	60.5
tr|J3KSS9|J3KSS9_HUMAN	P16849	22.44	303	198	10	57	337	36	323	2.00E-10	60.8
tr|J3KSS9|J3KSS9_HUMAN	Q83207	22.02	327	228	7	14	327	2	314	3.00E-10	60.1
tr|J3KSS9|J3KSS9_HUMAN	Q98146	24.54	326	213	9	28	338	25	332	5.00E-10	59.7
tr|J3KSS9|J3KSS9_HUMAN	Q6SW98	22.45	294	208	7	57	337	37	323	1.00E-09	58.5
tr|J3KSS9|J3KSS9_HUMAN	Q9J5I0	25.11	227	151	9	57	274	22	238	4.00E-09	56.6
tr|J3KSS9|J3KSS9_HUMAN	O12000	23.49	281	191	7	61	327	42	312	4.00E-09	56.6
tr|J3KSS9|J3KSS9_HUMAN	Q01035	22.42	281	203	7	55	331	42	311	1.00E	46.2

										-05	
tr|J3KSS9|J3KSS9_HUMAN	Q18LE5	26.88	93	67	1	58	149	29	121	3.00E-04	41.6
tr|J3KSS9|J3KSS9_HUMAN	Q66673	20.89	225	167	3	118	337	112	330	0.17	32.7
tr|J3KSS9|J3KSS9_HUMAN	Q2Y0E7	31.67	60	39	2	59	116	433	492	1	30.8
tr|J3KSS9|J3KSS9_HUMAN	Q65900	41.18	34	20	0	18	51	1357	1390	8.7	27.7
tr|J3KTN5|J3KTN5_HUMAN	Q08520	36.22	196	116	6	9	199	73	264	2.00E-33	124
tr|J3KTN5|J3KTN5_HUMAN	Q89609	36.32	190	116	3	13	199	90	277	3.00E-26	103
tr|J3KTN5|J3KTN5_HUMAN	P32229	33.13	166	103	5	39	199	1	163	2.00E-24	97.4
tr|J3KTN5|J3KTN5_HUMAN	Q86917	32.84	204	124	8	1	199	97	292	2.00E-22	92.8
tr|J3KTN5|J3KTN5_HUMAN	F5HF62	26.37	201	134	3	1	199	39	227	5.00E-17	77.4
tr|J3KTN5|J3KTN5_HUMAN	P69333	26.37	201	134	3	1	199	39	227	5.00E-17	77.4
tr|J3KTN5|J3KTN5_HUMAN	P69332	26.37	201	134	3	1	199	39	227	5.00E-17	77.4
tr|J3KTN5|J3KTN5_HUMAN	F5HDK1	31.38	188	114	8	4	185	41	219	2.00E-16	75.9
tr|J3KTN5|J3KTN5_HUMAN	P09703	31.38	188	114	8	4	185	39	217	4.00E-16	75.1
tr|J3KTN5|J3KTN5_HUMAN	Q9J5H4	27.98	193	125	7	1	184	33	220	1.00E-14	70.5
tr|J3KTN5|J3KTN5_HUMAN	Q9J529	25.3	166	110	6	33	190	54	213	1.00E-09	55.5
tr|J3KTN5|J3KTN5_HUMAN	Q9J5I0	25.93	189	124	8	1	180	23	204	1.00E-07	50.1

tr|J3KTN5|J3KTN5_HUMAN	Q18LE5	26.88	93	67	1	1	92	29	121	1.00E-04	41.2
tr|J3KTN5|J3KTN5_HUMAN	P16849	29.5	139	82	6	3	134	39	168	8.00E-04	38.9
tr|J3KTN5|J3KTN5_HUMAN	P52381	21.74	184	133	5	21	199	71	248	0.003	37
tr|J3KTN5|J3KTN5_HUMAN	Q83207	27.45	102	72	1	3	102	41	142	0.005	36.2
tr|J3KTN5|J3KTN5_HUMAN	O12000	25.68	148	97	3	4	145	42	182	0.006	36.2
tr|J3KTN5|J3KTN5_HUMAN	Q6SW98	23.81	210	141	6	3	199	40	243	0.007	35.8
tr|J3KTN5|J3KTN5_HUMAN	Q2Y0E7	31.67	60	39	2	2	59	433	492	0.95	29.6
sp|P32248|CCR7_HUMAN	Q89609	36.58	298	183	4	48	342	62	356	2.00E-46	165
sp|P32248|CCR7_HUMAN	Q08520	32.87	286	181	7	59	337	60	341	6.00E-45	160
sp|P32248|CCR7_HUMAN	F5HF62	30.21	341	223	4	40	377	15	343	2.00E-41	150
sp|P32248|CCR7_HUMAN	P69333	31.05	306	197	3	40	343	15	308	3.00E-40	147
sp|P32248|CCR7_HUMAN	P69332	31.05	306	197	3	40	343	15	308	3.00E-40	147
sp|P32248|CCR7_HUMAN	P32229	31.69	243	156	6	102	337	1	240	5.00E-36	133
sp|P32248|CCR7_HUMAN	Q86917	31.21	282	180	9	64	340	97	369	1.00E-31	123
sp|P32248|CCR7_HUMAN	F5HDK1	29.32	324	195	12	36	351	24	321	9.00E-24	100

sp|P32248|CCR7_HUMAN	P09703	28.47	295	187	10	67	351	39	319	2.00E-21	93.2
sp|P32248|CCR7_HUMAN	Q9J5H4	25.18	278	188	10	61	329	30	296	3.00E-17	81.3
sp|P32248|CCR7_HUMAN	Q9J529	23.27	245	172	7	96	330	54	292	2.00E-15	75.5
sp|P32248|CCR7_HUMAN	P52381	22.73	264	192	6	84	342	71	327	2.00E-10	61.2
sp|P32248|CCR7_HUMAN	P16849	22.44	303	198	10	63	343	36	323	2.00E-10	61.2
sp|P32248|CCR7_HUMAN	P52380	21.12	303	222	7	78	371	48	342	2.00E-10	60.8
sp|P32248|CCR7_HUMAN	Q83207	22.02	327	228	7	20	333	2	314	3.00E-10	60.5
sp|P32248|CCR7_HUMAN	Q98146	25.08	327	210	11	34	344	25	332	4.00E-10	59.7
sp|P32248|CCR7_HUMAN	Q6SW98	22.45	294	208	7	63	343	37	323	1.00E-09	58.5
sp|P32248|CCR7_HUMAN	O12000	23.49	281	191	7	67	333	42	312	5.00E-09	56.6
sp|P32248|CCR7_HUMAN	Q9J5I0	25.11	227	151	9	63	280	22	238	5.00E-09	56.6
sp|P32248|CCR7_HUMAN	Q01035	22.42	281	203	7	61	337	42	311	1.00E-05	45.8
sp|P32248|CCR7_HUMAN	Q18LE5	26.88	93	67	1	64	155	29	121	3.00E-04	41.6
sp|P32248|CCR7_HUMAN	Q66673	20.89	225	167	3	124	343	112	330	0.14	33.1
sp|P32248|CCR7_HUMAN	Q2Y0E7	31.67	60	39	2	65	122	433	492	0.95	30.8
sp|P32248|CCR7_HUMAN	Q65900	41.18	34	20	0	24	57	1357	1390	8.4	27.7
tr|C9JIP9|C9JIP9_HUMAN	O40955	27.12	59	31	2	5	51	1755	1813	4.4	26.2

tr|C9JIP9|C9JIP9_HUMAN	Q9J6K9	27.12	59	31	2	5	51	1755	1813	4.4	26.2
tr|C9JIP9|C9JIP9_HUMAN	Q99IE5	27.12	59	31	2	5	51	1755	1813	4.5	26.2
tr|C9JIP9|C9JIP9_HUMAN	P13889	27.12	59	31	2	5	51	1755	1813	4.6	26.2
tr|C9JIP9|C9JIP9_HUMAN	Q99IE7	27.12	59	31	2	5	51	1755	1813	4.6	26.2
tr|C9JIP9|C9JIP9_HUMAN	Q6X2U4	27.12	59	31	2	5	51	1755	1813	4.6	26.2
tr|C9JIP9|C9JIP9_HUMAN	Q6X2U2	27.12	59	31	2	5	51	1755	1813	4.7	26.2
tr|C9JIP9|C9JIP9_HUMAN	Q8BCR0	27.12	59	31	2	5	51	1755	1813	4.8	26.2
tr|C9JIP9|C9JIP9_HUMAN	P16493	28.57	42	30	0	2	43	672	713	4.9	26.2
tr|C9JIP9|C9JIP9_HUMAN	Q86500	27.12	59	31	2	5	51	1755	1813	4.9	26.2
tr|C9JIP9|C9JIP9_HUMAN	Q66478	32.14	28	19	0	43	70	479	506	6.7	25.8
tr|C9JIP9|C9JIP9_HUMAN	Q66479	32.14	28	19	0	43	70	479	506	7	25.4
tr|C9JIP9|C9JIP9_HUMAN	P14360	35.9	39	21	1	5	39	52	90	8.3	25
sp|P51685|CCR8_HUMAN	Q86917	44.14	290	153	5	25	311	83	366	1.00E-79	251
sp|P51685|CCR8_HUMAN	Q08520	36.88	320	186	7	10	314	26	344	7.00E-65	212
sp|P51685|CCR8_HUMAN	Q89609	38.95	267	160	2	53	316	90	356	4.00E-60	200
sp|P51685|CCR8_HUMAN	P32229	37.55	253	147	5	79	324	1	249	5.00E-55	183
sp|P51685|CCR8_HUMAN	F5HF62	29.93	294	197	4	19	311	17	302	1.00E-45	161
sp|P51685|CCR8_HUMAN	P69333	28.99	307	209	4	19	324	17	315	2.00E-45	160
sp|P51685|CCR8_HUMAN	P69332	28.99	307	209	4	19	324	17	315	2.00E-45	160
sp|P51685|CCR8_HUMAN	F5HDK	27.2	261	185	3	53	311	50	307	2.00E	102

	1									-24	
sp|P51685|CCR8_HUMAN	P09703	26.82	261	186	3	53	311	48	305	3.00E-24	101
sp|P51685|CCR8_HUMAN	Q9J5H4	26.26	278	188	7	35	303	27	296	6.00E-21	91.3
sp|P51685|CCR8_HUMAN	Q9J5I0	25.09	279	191	8	34	305	16	283	3.00E-15	75.1
sp|P51685|CCR8_HUMAN	Q9J529	24.83	290	202	9	35	313	17	301	8.00E-14	70.5
sp|P51685|CCR8_HUMAN	Q83207	23.96	288	203	7	68	346	68	348	3.00E-12	66.6
sp|P51685|CCR8_HUMAN	O12000	21.43	252	185	6	68	310	68	315	5.00E-11	62.8
sp|P51685|CCR8_HUMAN	Q6SW98	19.94	351	226	14	44	353	42	378	5.00E-10	59.7
sp|P51685|CCR8_HUMAN	P16849	19.51	328	227	12	44	348	41	354	7.00E-10	59.3
sp|P51685|CCR8_HUMAN	Q98146	22.74	277	210	3	44	317	56	331	2.00E-09	57.4
sp|P51685|CCR8_HUMAN	Q01035	20.66	334	235	11	1	319	1	319	6.00E-09	55.8
sp|P51685|CCR8_HUMAN	P52380	19.37	284	215	6	70	350	65	337	2.00E-05	45.1
sp|P51685|CCR8_HUMAN	Q66673	23.08	286	213	6	35	317	49	330	0.002	38.9
sp|P51685|CCR8_HUMAN	P52381	21.29	249	182	8	67	308	78	319	0.007	37.4
sp|P51685|CCR8_HUMAN	Q5UQE9	26.74	86	58	3	260	342	36	119	2.5	29.3
sp|P51685|CCR8_HUMAN	Q18LE5	23.88	134	86	5	98	227	87	208	3.8	28.5
sp|P51685|CCR8_HUMAN	Q66431	34.62	52	32	2	177	228	1465	1514	6.4	28.1
sp|P51685|CCR8_HUMAN	Q5UNV	25.81	93	57	3	271	355	171	259	7	27.7

	7										
tr|C9JWC0|C9JWC0_HUMAN	P0C6Y0	42.31	26	13	1	82	107	410	433	2.4	26.6
tr|C9JWC0|C9JWC0_HUMAN	P0C6V1	42.31	26	13	1	82	107	410	433	3	26.2
tr|C9JWC0|C9JWC0_HUMAN	Q69140	50	20	10	0	87	106	706	725	4.7	25.8
tr|C9JWC0|C9JWC0_HUMAN	P36216	47.62	21	11	0	54	74	228	248	6.6	25
tr|C9JWC0|C9JWC0_HUMAN	P27315	30.3	33	23	0	5	37	945	977	6.7	25.4
tr|C9JWC0|C9JWC0_HUMAN	P41264	29.11	79	53	2	8	86	137	212	6.8	25
tr|C9JWC0|C9JWC0_HUMAN	Q83887	33.33	27	18	0	5	31	943	969	9	25
sp|P51686|CCR9_HUMAN	Q89609	34.48	319	204	3	38	354	62	377	2.00E-49	172
sp|P51686|CCR9_HUMAN	Q08520	27.24	279	191	7	54	325	65	338	9.00E-36	135
sp|P51686|CCR9_HUMAN	F5HF62	28.73	362	216	10	16	368	15	343	5.00E-33	127
sp|P51686|CCR9_HUMAN	P69333	29.43	316	192	8	16	328	15	302	2.00E-32	125
sp|P51686|CCR9_HUMAN	P69332	29.43	316	192	8	16	328	15	302	2.00E-32	125
sp|P51686|CCR9_HUMAN	Q86917	26.96	293	203	8	51	339	94	379	9.00E-32	123
sp|P51686|CCR9_HUMAN	P32229	26.56	241	166	6	92	325	1	237	8.00E-28	110
sp|P51686|CCR9_HUMAN	F5HDK	28.57	294	199	6	56	346	40	325	4.00E	110

	1									-27	
sp|P51686|CCR9_HUMAN	P09703	27.89	294	201	6	56	346	38	323	6.00E-26	106
sp|P51686|CCR9_HUMAN	Q9J5H4	24.01	279	191	10	51	320	30	296	6.00E-20	88.6
sp|P51686|CCR9_HUMAN	Q9J5I0	25.96	312	209	9	54	358	23	319	9.00E-17	79.3
sp|P51686|CCR9_HUMAN	Q9J529	23.91	276	192	7	57	322	26	293	3.00E-16	77.8
sp|P51686|CCR9_HUMAN	O12000	24	325	221	12	18	329	6	317	2.00E-13	70.1
sp|P51686|CCR9_HUMAN	Q83207	22.09	326	230	8	18	330	6	320	2.00E-11	63.9
sp|P51686|CCR9_HUMAN	Q6SW98	21.95	287	192	11	56	324	41	313	2.00E-10	60.8
sp|P51686|CCR9_HUMAN	Q98146	22.66	353	234	9	1	335	1	332	7.00E-10	59.3
sp|P51686|CCR9_HUMAN	P16849	21.03	290	192	12	56	324	40	313	1.00E-09	58.5
sp|P51686|CCR9_HUMAN	Q01035	22.12	321	231	7	24	335	8	318	7.00E-08	52.8
sp|P51686|CCR9_HUMAN	P52381	22.01	259	191	5	70	325	69	319	1.00E-07	52.4
sp|P51686|CCR9_HUMAN	P52380	22.31	251	174	6	83	325	65	302	6.00E-05	43.9
sp|P51686|CCR9_HUMAN	Q18LE5	22.22	234	162	7	42	270	17	235	6.00E-04	40.8
sp|P51686|CCR9_HUMAN	P0C6Y2	23.36	137	90	3	226	350	4891	5024	0.088	34.3
sp|P51686|CCR9_HUMAN	P0C6Y1	23.36	137	90	3	226	350	4891	5024	0.089	34.3
sp|P51686|CCR9_HUMAN	P0C6Y3	23.36	137	90	3	226	350	4893	5026	0.089	34.3

sp|P51686|CCR9_HUMAN	O10438	42.42	33	17	1	322	354	24	54	4.2	28.5
sp|P51686|CCR9_HUMAN	A4KZ49	28.79	66	37	2	20	85	1137	1192	8.2	27.7
sp|P51686|CCR9_HUMAN	Q306W8	28.57	56	40	0	213	268	398	453	8.9	27.7
sp|Q9NPB9|ACKR4_HUMAN	F5HF62	28.66	307	211	5	22	328	14	312	2.00E-37	139
sp|Q9NPB9|ACKR4_HUMAN	P69333	28.66	307	211	5	22	328	14	312	4.00E-37	138
sp|Q9NPB9|ACKR4_HUMAN	P69332	28.66	307	211	5	22	328	14	312	4.00E-37	138
sp|Q9NPB9|ACKR4_HUMAN	Q89609	30.77	325	210	8	31	345	62	381	7.00E-37	137
sp|Q9NPB9|ACKR4_HUMAN	Q08520	27.64	351	215	10	4	331	24	358	4.00E-31	121
sp|Q9NPB9|ACKR4_HUMAN	Q86917	26.32	285	176	9	51	317	101	369	7.00E-26	105
sp|Q9NPB9|ACKR4_HUMAN	Q9J5H4	24.51	306	199	11	16	306	8	296	3.00E-21	92
sp|Q9NPB9|ACKR4_HUMAN	P32229	26.95	256	153	7	85	318	1	244	2.00E-20	88.6
sp|Q9NPB9|ACKR4_HUMAN	F5HDK1	27.27	275	170	10	53	311	44	304	1.00E-18	84.7
sp|Q9NPB9|ACKR4_HUMAN	P09703	26.91	275	171	10	53	311	42	302	1.00E-18	84.7
sp|Q9NPB9|ACKR4_HUMAN	Q9J529	25.36	280	188	10	44	308	20	293	2.00E-15	75.1
sp|Q9NPB9|ACKR4_HUMAN	O12000	24.45	274	196	5	78	341	72	344	8.00E-14	71.2
sp|Q9NPB9|ACKR4_HUMAN	Q9J5I0	26.17	321	213	12	25	333	3	311	5.00E-13	68.2

sp|Q9NPB9|ACKR4_HUMAN	Q83207	22.46	334	244	6	10	329	2	334	1.00E-12	67.4
sp|Q9NPB9|ACKR4_HUMAN	P16849	23.79	248	170	6	78	310	70	313	1.00E-09	58.5
sp|Q9NPB9|ACKR4_HUMAN	Q6SW98	24.9	253	160	8	78	310	71	313	1.00E-08	55.1
sp|Q9NPB9|ACKR4_HUMAN	P52380	22.13	253	175	7	72	312	61	303	3.00E-07	50.8
sp|Q9NPB9|ACKR4_HUMAN	Q98146	19.45	293	215	7	48	330	54	335	3.00E-06	47.4
sp|Q9NPB9|ACKR4_HUMAN	P52381	19.68	310	228	8	12	311	21	319	9.00E-05	43.1
sp|Q9NPB9|ACKR4_HUMAN	Q01035	18.35	267	209	5	50	312	48	309	5.00E-04	40.4
sp|Q9NPB9|ACKR4_HUMAN	Q18LE5	20.95	105	82	1	35	138	17	121	0.027	35.4
sp|Q9NPB9|ACKR4_HUMAN	P41423	38.1	21	13	0	256	276	71	91	5	28.1
sp|Q9NPB9|ACKR4_HUMAN	Q88898	43.9	41	18	2	147	187	313	348	7.6	27.7
sp|Q9NPB9|ACKR4_HUMAN	Q91FV8	34.78	23	15	0	316	338	377	399	8.8	27.3
tr|C9JP23|C9JP23_HUMAN	Q89609	39.5	200	107	4	25	215	59	253	7.00E-27	105
tr|C9JP23|C9JP23_HUMAN	Q86917	27.94	204	132	6	19	215	74	269	3.00E-20	87.4
tr|C9JP23|C9JP23_HUMAN	Q08520	27.37	179	114	6	48	216	69	241	7.00E-16	74.7
tr|C9JP23|C9JP23_HUMAN	F5HF62	24.42	172	114	4	19	179	13	179	9.00E-09	53.5

tr|C9JP23|C9JP23_HUMAN	P69333	24.42	172	114	4	19	179	13	179	9.00E-09	53.5
tr|C9JP23|C9JP23_HUMAN	P69332	24.42	172	114	4	19	179	13	179	9.00E-09	53.5
tr|C9JP23|C9JP23_HUMAN	P32229	24.83	145	94	5	82	216	1	140	1.00E-08	52.8
tr|C9JP23|C9JP23_HUMAN	P09703	24.14	174	112	6	50	213	42	205	8.00E-07	48.1
tr|C9JP23|C9JP23_HUMAN	F5HDK1	24.14	174	112	6	50	213	44	207	2.00E-06	47
tr|C9JP23|C9JP23_HUMAN	Q9J5I0	28.09	89	55	3	49	129	28	115	0.004	36.6
tr|C9JP23|C9JP23_HUMAN	Q9J5H4	22.32	112	75	4	27	130	19	126	0.02	34.7
tr|C9JP23|C9JP23_HUMAN	P16849	26.56	128	84	4	48	168	42	166	0.023	34.7
tr|C9JP23|C9JP23_HUMAN	Q6SW98	25.73	171	108	6	48	202	43	210	0.03	34.3
tr|C9JP23|C9JP23_HUMAN	Q98146	22.45	147	92	3	6	131	1	146	0.031	33.9
tr|C9JP23|C9JP23_HUMAN	Q9J529	24.35	115	77	3	74	180	52	164	0.031	33.9
tr|C9JP23|C9JP23_HUMAN	Q05221	38.46	39	24	0	100	138	137	175	0.15	32
tr|C9JP23|C9JP23_HUMAN	Q18LE5	21.67	120	87	1	23	135	8	127	0.3	31.2
tr|C9JP23|C9JP23_HUMAN	P0C6V2	40.54	37	20	1	154	190	1765	1799	1.9	28.9
tr|C9JP23|C9JP23_HUMAN	P0C6Y5	40.54	37	20	1	154	190	1765	1799	2.2	28.5
tr|C9JP23|C9JP23_HUMAN	F5HET1	25.76	66	47	1	102	165	108	173	3.8	27.7
tr|C9JP23|C9JP23_HUMAN	P16751	25.76	66	47	1	102	165	108	173	3.8	27.7
tr|C9JP23|C9JP23_HUMAN	Q83207	20.83	168	116	6	13	165	2	167	4	27.7
tr|C9JP23|C9JP23_HUMAN	P0C6T4	38.3	47	29	0	9	55	1005	1051	9.4	26.6
tr|C9JP23|C9JP23_HUMAN	O12000	21.55	116	83	3	74	183	71	184	9.6	26.6
sp|O00421|CCRL2_HUMAN	Q89609	38.91	329	178	8	25	335	59	382	1.00E-53	183
sp|O00421|CCRL2_HUMA	Q86917	28.05	303	202	7	19	313	74	368	6.00E	132

N										-35	
sp|O00421|CCRL2_HUMAN	Q08520	26.94	271	177	9	48	303	69	333	4.00E-26	106
sp|O00421|CCRL2_HUMAN	F5HF62	23.78	307	203	7	19	311	13	302	2.00E-25	104
sp|O00421|CCRL2_HUMAN	P69333	23.78	307	203	7	19	311	13	302	3.00E-25	103
sp|O00421|CCRL2_HUMAN	P69332	23.78	307	203	7	19	311	13	302	3.00E-25	103
sp|O00421|CCRL2_HUMAN	P32229	25.32	237	157	8	82	303	1	232	3.00E-19	85.1
sp|O00421|CCRL2_HUMAN	Q6SW98	23.86	285	194	9	48	315	43	321	1.00E-12	67.8
sp|O00421|CCRL2_HUMAN	F5HDK1	20.83	312	215	9	50	341	44	343	3.00E-11	63.5
sp|O00421|CCRL2_HUMAN	P09703	20.83	312	215	9	50	341	42	341	3.00E-11	63.2
sp|O00421|CCRL2_HUMAN	P16849	22.03	286	199	9	48	315	42	321	1.00E-10	61.6
sp|O00421|CCRL2_HUMAN	Q83207	22.7	326	224	13	13	315	2	322	1.00E-08	55.5
sp|O00421|CCRL2_HUMAN	O12000	20.72	251	187	5	74	314	71	319	8.00E-08	52.8
sp|O00421|CCRL2_HUMAN	Q9J529	22.92	253	169	9	75	309	53	297	3.00E-07	50.4
sp|O00421|CCRL2_HUMAN	Q98146	19.64	336	225	10	6	309	1	323	6.00E-05	43.5
sp|O00421|CCRL2_HUMAN	P52381	22.29	332	219	11	1	307	1	318	6.00E-04	40.4
sp|O00421|CCRL2_HUMA	Q9J5I0	20.92	239	161	9	49	271	28	254	7.00E	40.4

N										-04	
sp|O00421|CCRL2_HUMAN	P52380	29.13	127	82	4	196	316	186	310	0.003	38.5
sp|O00421|CCRL2_HUMAN	Q9J5H4	19.4	299	212	10	27	310	19	303	0.004	37.7
sp|O00421|CCRL2_HUMAN	Q05221	38.46	39	24	0	100	138	137	175	0.28	32
sp|O00421|CCRL2_HUMAN	Q18LE5	21.67	120	87	1	23	135	8	127	0.38	31.6
sp|O00421|CCRL2_HUMAN	P0C6V2	40.54	37	20	1	154	190	1765	1799	4	28.9
sp|O00421|CCRL2_HUMAN	P0C6Y5	40.54	37	20	1	154	190	1765	1799	4.2	28.9
sp|O00421|CCRL2_HUMAN	F5HET1	25.76	66	47	1	102	165	108	173	5.8	28.1
sp|O00421|CCRL2_HUMAN	P16751	25.76	66	47	1	102	165	108	173	5.8	28.1
tr|C9JLM2|C9JLM2_HUMAN	P69333	36.46	96	61	0	11	106	13	108	1.00E-16	73.6
tr|C9JLM2|C9JLM2_HUMAN	P69332	36.46	96	61	0	11	106	13	108	1.00E-16	73.6
tr|C9JLM2|C9JLM2_HUMAN	F5HF62	36.84	95	60	0	12	106	14	108	3.00E-16	72.4
tr|C9JLM2|C9JLM2_HUMAN	Q86917	41.98	81	42	3	25	104	88	164	7.00E-09	51.6
tr|C9JLM2|C9JLM2_HUMAN	Q08520	36.62	71	44	1	36	106	64	133	9.00E-09	51.2
tr|C9JLM2|C9JLM2_HUMAN	Q9J5I0	38.46	65	39	1	28	92	14	77	4.00E-06	43.1
tr|C9JLM2|C9JLM2_HUMA	Q9J5H4	31.82	66	44	1	29	94	25	89	1.00E	42

N										-05	
tr|C9JLM2|C9JLM2_HUMAN	P09703	38.89	54	29	1	41	90	37	90	1.00E-04	38.5
tr|C9JLM2|C9JLM2_HUMAN	Q89609	55.81	43	19	0	49	91	90	132	2.00E-04	38.1
tr|C9JLM2|C9JLM2_HUMAN	F5HDK1	43.75	48	26	1	44	90	45	92	2.00E-04	38.1
tr|C9JLM2|C9JLM2_HUMAN	O12000	40.62	64	30	3	39	97	38	98	0.01	33.1
tr|C9JLM2|C9JLM2_HUMAN	Q9J529	30.99	71	39	3	22	85	3	70	0.23	29.3
tr|C9JLM2|C9JLM2_HUMAN	Q98146	32.61	46	31	0	39	84	55	100	1.4	26.9
tr|C9JLM2|C9JLM2_HUMAN	Q67704	28.26	46	27	1	4	43	1500	1545	1.4	27.3
tr|C9JLM2|C9JLM2_HUMAN	Q2YHF2	32.65	49	32	1	28	75	718	766	1.4	27.3
tr|C9JLM2|C9JLM2_HUMAN	Q83207	36	50	30	1	39	86	41	90	1.6	26.9
tr|C9JLM2|C9JLM2_HUMAN	Q2YHF0	36.36	33	21	0	28	60	718	750	2.9	26.2
tr|C9JLM2|C9JLM2_HUMAN	P03304	27.78	72	42	3	10	81	2149	2210	3.4	26.2
tr|C9JLM2|C9JLM2_HUMAN	Q66765	27.78	72	42	3	10	81	2151	2212	3.4	26.2
tr|C9JLM2|C9JLM2_HUMAN	Q88668	36.36	33	21	0	28	60	718	750	5.6	25.4
tr|C9JLM2|C9JLM2_HUMAN	P24911	29.82	57	38	1	19	73	122	178	8.3	25
tr|C9JLM2|C9JLM2_HUMA	Q65165	32	50	31	1	47	93	291	340	8.9	25

N											
tr|C9JLM2|C9JLM2_HUMAN	Q5UCB8	33.33	33	22	0	28	60	718	750	9.1	25
tr|C9JLM2|C9JLM2_HUMAN	Q58HT7	33.33	33	22	0	28	60	718	750	9.1	25
tr|C9JN40|C9JN40_HUMAN	F5HF62	37.82	156	93	1	11	166	13	164	4.00E-31	115
tr|C9JN40|C9JN40_HUMAN	P69333	37.82	156	93	1	11	166	13	164	5.00E-31	115
tr|C9JN40|C9JN40_HUMAN	P69332	37.82	156	93	1	11	166	13	164	5.00E-31	115
tr|C9JN40|C9JN40_HUMAN	Q86917	42.14	140	76	3	25	163	88	223	6.00E-27	104
tr|C9JN40|C9JN40_HUMAN	Q08520	36.29	124	78	1	40	163	68	190	9.00E-24	95.5
tr|C9JN40|C9JN40_HUMAN	Q89609	34.42	154	91	2	18	165	59	208	3.00E-16	74.3
tr|C9JN40|C9JN40_HUMAN	P32229	31.46	89	61	0	75	163	1	89	2.00E-13	65.5
tr|C9JN40|C9JN40_HUMAN	P09703	29.77	131	87	2	41	166	37	167	8.00E-13	64.7
tr|C9JN40|C9JN40_HUMAN	F5HDK1	31.2	125	84	2	44	166	45	169	6.00E-12	62
tr|C9JN40|C9JN40_HUMAN	Q9J5I0	39.05	105	57	3	28	128	14	115	1.00E-11	60.8
tr|C9JN40|C9JN40_HUMAN	Q9J5H4	32.38	105	68	2	29	131	25	128	4.00E-11	59.7
tr|C9JN40|C9JN40_HUMAN	Q9J529	30.47	128	74	6	24	141	5	127	1.00E-05	43.5
tr|C9JN40|C9JN40_HUMA	O12000	29.32	133	82	5	39	163	38	166	1.00E	40.4

N										-04	
tr|C9JN40|C9JN40_HUMAN	Q98146	25.4	126	83	3	39	161	55	172	4.00E-04	38.9
tr|C9JN40|C9JN40_HUMAN	Q18LE5	22.13	122	90	3	34	151	27	147	0.007	35.4
tr|C9JN40|C9JN40_HUMAN	Q83207	23.85	130	91	3	39	163	41	167	0.01	35
tr|C9JN40|C9JN40_HUMAN	P16849	23.95	167	119	5	1	163	1	163	0.25	30.8
tr|C9JN40|C9JN40_HUMAN	Q6SW98	26.72	116	79	3	50	163	53	164	2.6	27.7
tr|C9JN40|C9JN40_HUMAN	Q67704	28.26	46	27	1	4	43	1500	1545	3.9	27.3
tr|C9JN40|C9JN40_HUMAN	Q2YHF2	32.65	49	32	1	28	75	718	766	3.9	27.3
tr|C9JN40|C9JN40_HUMAN	Q01035	22.73	110	84	1	34	143	42	150	6.3	26.2
tr|C9JN40|C9JN40_HUMAN	Q6R7K0	29.29	99	64	5	47	143	839	933	6.3	26.6
tr|C9JN40|C9JN40_HUMAN	Q2YHF0	36.36	33	21	0	28	60	718	750	7.4	26.2
tr|C9JN40|C9JN40_HUMAN	Q66765	27.78	72	42	3	10	81	2151	2212	8.8	26.2
tr|C9JN40|C9JN40_HUMAN	P03304	27.78	72	42	3	10	81	2149	2210	9	26.2
sp|P49238|CX3C1_HUMAN	F5HF62	38.2	322	193	3	11	331	13	329	2.00E-75	239
sp|P49238|CX3C1_HUMAN	P69333	38.26	311	186	3	11	320	13	318	3.00E-74	236
sp|P49238|CX3C1_HUMAN	P69332	38.26	311	186	3	11	320	13	318	3.00E	236

										-74	
sp|P49238|CX3C1_HUMAN	Q89609	37.72	289	170	4	49	330	90	375	1.00E -59	199
sp|P49238|CX3C1_HUMAN	Q86917	38.89	288	165	6	25	307	88	369	3.00E -56	190
sp|P49238|CX3C1_HUMAN	Q08520	32.26	279	172	6	36	301	64	338	2.00E -47	166
sp|P49238|CX3C1_HUMAN	P32229	30.42	240	151	5	75	301	1	237	8.00E -37	135
sp|P49238|CX3C1_HUMAN	P09703	26.56	305	212	4	41	334	37	340	2.00E -33	128
sp|P49238|CX3C1_HUMAN	F5HDK 1	27.09	299	209	4	44	334	45	342	2.00E -33	128
sp|P49238|CX3C1_HUMAN	Q9J5H4	23.57	280	194	10	29	296	25	296	2.00E -18	84
sp|P49238|CX3C1_HUMAN	Q9J5I0	26.57	286	179	11	28	298	14	283	1.00E -16	79
sp|P49238|CX3C1_HUMAN	Q9J529	25.58	301	190	13	22	298	3	293	9.00E -14	70.5
sp|P49238|CX3C1_HUMAN	Q83207	21.03	290	206	7	39	310	41	325	3.00E -12	66.2
sp|P49238|CX3C1_HUMAN	O12000	22.71	317	207	10	39	335	38	336	7.00E -12	65.5
sp|P49238|CX3C1_HUMAN	Q98146	24.64	276	201	3	39	308	55	329	9.00E -12	64.7
sp|P49238|CX3C1_HUMAN	Q01035	24.07	270	202	3	34	302	42	309	2.00E -11	63.9
sp|P49238|CX3C1_HUMAN	P16849	20.82	317	230	7	1	300	1	313	6.00E -09	56.2
sp|P49238|CX3C1_HUMAN	P52381	24.54	269	181	9	63	320	78	335	3.00E	53.9

										-08	
sp|P49238|CX3C1_HUMAN	Q6SW98	23.3	279	189	8	40	300	42	313	3.00E-07	50.8
sp|P49238|CX3C1_HUMAN	Q66673	25.38	327	212	11	4	310	16	330	1.00E-05	45.8
sp|P49238|CX3C1_HUMAN	P52380	21.8	289	208	9	64	341	63	344	8.00E-05	43.1
sp|P49238|CX3C1_HUMAN	Q18LE5	22.13	122	90	3	34	151	27	147	0.019	35.8
sp|P25024|CXCR1_HUMAN	F5HF62	33.99	303	183	6	24	321	17	307	1.00E-43	155
sp|P25024|CXCR1_HUMAN	P69333	33.99	303	183	6	24	321	17	307	2.00E-43	155
sp|P25024|CXCR1_HUMAN	P69332	33.99	303	183	6	24	321	17	307	2.00E-43	155
sp|P25024|CXCR1_HUMAN	Q08520	29.47	319	212	8	2	313	26	338	2.00E-43	155
sp|P25024|CXCR1_HUMAN	Q89609	33.72	261	162	7	59	313	93	348	3.00E-35	133
sp|P25024|CXCR1_HUMAN	Q86917	30.28	284	184	9	42	319	94	369	1.00E-33	129
sp|P25024|CXCR1_HUMAN	P32229	28.57	238	162	5	83	313	1	237	1.00E-31	120
sp|P25024|CXCR1_HUMAN	Q98146	28.89	315	218	4	4	314	11	323	3.00E-28	112
sp|P25024|CXCR1_HUMAN	Q01035	28.23	294	195	6	26	314	27	309	8.00E-26	105
sp|P25024|CXCR1_HUMAN	Q9J5H4	24.5	298	199	9	24	308	12	296	2.00E-25	104
sp|P25024|CXCR1_HUMAN	Q9J5I0	28.42	292	194	6	24	310	2	283	2.00E-19	87

sp|P25024|CXCR1_HUMAN	F5HDK1	28	275	179	10	49	313	39	304	5.00E-17	80.5
sp|P25024|CXCR1_HUMAN	Q9J529	24.28	313	200	11	15	310	1	293	2.00E-16	78.2
sp|P25024|CXCR1_HUMAN	P09703	27.9	276	178	11	49	313	37	302	2.00E-16	78.6
sp|P25024|CXCR1_HUMAN	O12000	24.67	304	207	10	47	335	38	334	6.00E-10	59.3
sp|P25024|CXCR1_HUMAN	Q83207	23.97	267	190	7	64	320	59	322	3.00E-08	53.9
sp|P25024|CXCR1_HUMAN	Q6SW98	22.91	275	182	12	57	312	50	313	6.00E-08	53.1
sp|P25024|CXCR1_HUMAN	P16849	23.91	276	179	13	57	312	49	313	6.00E-08	53.1
sp|P25024|CXCR1_HUMAN	Q66673	29.79	94	66	0	222	315	230	323	3.00E-05	44.3
sp|P25024|CXCR1_HUMAN	P52381	21.71	304	220	8	24	316	26	322	1.00E-04	42.7
sp|P25024|CXCR1_HUMAN	P52380	20.61	262	199	5	59	316	49	305	5.00E-04	40.8
sp|P25024|CXCR1_HUMAN	Q18LE5	23.58	106	75	3	34	136	19	121	0.02	35.8
sp|P25024|CXCR1_HUMAN	Q5UPK5	24.32	74	56	0	2	75	16	89	4.4	28.1
sp|P25024|CXCR1_HUMAN	Q8JJX1	34.15	41	22	1	185	225	227	262	4.7	28.5
sp|P25024|CXCR1_HUMAN	Q8QL53	34.15	41	22	1	185	225	227	262	5	28.5
sp|P25024|CXCR1_HUMAN	O36364	29.49	78	50	3	88	162	58	133	5.8	27.7

sp|P25024|CXCR1_HUMAN	Q02270	22.14	131	88	3	53	170	21	150	8	26.9
tr|C9J1J7|C9J1J7_HUMAN	Q08520	38.46	130	78	2	44	172	55	183	3.00E-25	99.8
tr|C9J1J7|C9J1J7_HUMAN	Q86917	31.3	131	85	4	33	160	77	205	5.00E-16	74.3
tr|C9J1J7|C9J1J7_HUMAN	F5HF62	34.19	117	76	1	35	150	19	135	1.00E-13	67
tr|C9J1J7|C9J1J7_HUMAN	P69333	34.19	117	76	1	35	150	19	135	2.00E-13	66.6
tr|C9J1J7|C9J1J7_HUMAN	P69332	34.19	117	76	1	35	150	19	135	2.00E-13	66.6
tr|C9J1J7|C9J1J7_HUMAN	Q9J5H4	33.94	109	62	3	53	159	32	132	1.00E-12	63.9
tr|C9J1J7|C9J1J7_HUMAN	P32229	36.59	82	51	1	92	172	1	82	2.00E-12	62.4
tr|C9J1J7|C9J1J7_HUMAN	Q9J5I0	37.11	97	58	2	51	145	20	115	5.00E-12	62
tr|C9J1J7|C9J1J7_HUMAN	Q01035	31.61	155	103	3	15	168	7	159	7.00E-09	52.8
tr|C9J1J7|C9J1J7_HUMAN	Q89609	36.11	108	65	3	68	172	93	199	2.00E-08	51.6
tr|C9J1J7|C9J1J7_HUMAN	Q98146	27.95	161	111	3	2	161	4	160	6.00E-08	50.1
tr|C9J1J7|C9J1J7_HUMAN	Q9J529	28.8	125	77	4	53	170	22	141	1.00E-07	49.7
tr|C9J1J7|C9J1J7_HUMAN	F5HDK1	31.82	110	67	3	58	159	39	148	1.00E-06	46.2
tr|C9J1J7|C9J1J7_HUMAN	P09703	30.91	110	68	3	58	159	37	146	7.00E-06	44.3

tr|C9J1J7|C9J1J7_HUMAN	Q18LE5	23.23	99	71	2	50	145	25	121	0.004	36.2
tr|C9J1J7|C9J1J7_HUMAN	O12000	33.02	106	62	4	56	154	38	141	0.011	35
tr|C9J1J7|C9J1J7_HUMAN	P16849	25.17	147	103	5	24	167	8	150	0.035	33.5
tr|C9J1J7|C9J1J7_HUMAN	Q83207	29.06	117	80	3	39	152	23	139	0.048	32.7
tr|C9J1J7|C9J1J7_HUMAN	Q6SW98	29.25	106	67	6	66	167	50	151	0.39	30
tr|C9J1J7|C9J1J7_HUMAN	Q5I147	29.79	47	33	0	104	150	144	190	5.4	26.6
tr|C9J1J7|C9J1J7_HUMAN	P06501	29.17	48	34	0	123	170	76	123	6.7	26.2
tr|C9J2F9|C9J2F9_HUMAN	Q08520	38.95	95	57	1	44	138	55	148	1.00E-18	80.5
tr|C9J2F9|C9J2F9_HUMAN	F5HF62	43.66	71	39	1	35	104	19	89	3.00E-10	56.6
tr|C9J2F9|C9J2F9_HUMAN	P69333	43.66	71	39	1	35	104	19	89	3.00E-10	56.2
tr|C9J2F9|C9J2F9_HUMAN	P69332	43.66	71	39	1	35	104	19	89	3.00E-10	56.2
tr|C9J2F9|C9J2F9_HUMAN	Q9J5H4	31.82	88	57	2	53	138	32	118	7.00E-09	52.4
tr|C9J2F9|C9J2F9_HUMAN	Q86917	29.13	103	70	3	33	134	77	177	9.00E-09	52
tr|C9J2F9|C9J2F9_HUMAN	Q9J5I0	34.44	90	56	2	51	138	20	108	1.00E-08	51.6
tr|C9J2F9|C9J2F9_HUMAN	P32229	36.17	47	30	0	92	138	1	47	5.00E-06	43.5
tr|C9J2F9|C9J2F9_HUMAN	Q01035	34.52	84	54	1	15	98	7	89	9.00E-06	42.7
tr|C9J2F9|C9J2F9_HUMAN	F5HDK1	44.9	49	23	1	58	102	39	87	3.00E-04	38.9
tr|C9J2F9|C9J2F9_HUMAN	Q9J529	30.68	88	50	3	53	134	22	104	7.00E-04	37.4

tr|C9J2F9|C9J2F9_HUMAN	P09703	42.86	49	24	1	58	102	37	85	0.001	36.6
tr|C9J2F9|C9J2F9_HUMAN	Q98146	29.7	101	66	3	2	101	4	100	0.003	35.8
tr|C9J2F9|C9J2F9_HUMAN	Q89609	37.5	72	42	2	68	137	93	163	0.003	35.8
tr|C9J2F9|C9J2F9_HUMAN	Q18LE5	23.86	88	62	2	50	134	25	110	0.026	33.1
tr|C9J2F9|C9J2F9_HUMAN	P08557	40	30	18	0	9	38	116	145	2.8	26.9
tr|C9JG19|C9JG19_HUMAN	Q08520	39.78	93	55	1	44	136	55	146	2.00E-18	79.7
tr|C9JG19|C9JG19_HUMAN	F5HF62	43.66	71	39	1	35	104	19	89	3.00E-10	56.6
tr|C9JG19|C9JG19_HUMAN	P69333	43.66	71	39	1	35	104	19	89	4.00E-10	56.2
tr|C9JG19|C9JG19_HUMAN	P69332	43.66	71	39	1	35	104	19	89	4.00E-10	56.2
tr|C9JG19|C9JG19_HUMAN	Q86917	29.13	103	70	3	33	134	77	177	1.00E-08	51.6
tr|C9JG19|C9JG19_HUMAN	Q9J5H4	31.4	86	56	2	53	136	32	116	2.00E-08	50.8
tr|C9JG19|C9JG19_HUMAN	Q9J5I0	34.09	88	55	2	51	136	20	106	4.00E-08	50.1
tr|C9JG19|C9JG19_HUMAN	Q01035	34.52	84	54	1	15	98	7	89	8.00E-06	43.1
tr|C9JG19|C9JG19_HUMAN	P32229	37.78	45	28	0	92	136	1	45	9.00E-06	42.7
tr|C9JG19|C9JG19_HUMAN	F5HDK1	44.9	49	23	1	58	102	39	87	2.00E-04	38.9
tr|C9JG19|C9JG19_HUMAN	Q9J529	30.68	88	50	3	53	134	22	104	7.00E-04	37.4
tr|C9JG19|C9JG19_HUMAN	P09703	42.86	49	24	1	58	102	37	85	0.001	36.6
tr|C9JG19|C9JG19_HUMA	Q98146	29.7	101	66	3	2	101	4	100	0.003	35.8

N											
tr|C9JG19|C9JG19_HUMAN	Q89609	39.13	69	39	2	68	134	93	160	0.003	35.4
tr|C9JG19|C9JG19_HUMAN	Q18LE5	23.86	88	62	2	50	134	25	110	0.024	33.1
tr|C9JG19|C9JG19_HUMAN	P08557	40	30	18	0	9	38	116	145	2.7	26.9
tr|C9JW47|C9JW47_HUMAN	Q08520	39.86	138	81	2	44	180	55	191	5.00E-28	108
tr|C9JW47|C9JW47_HUMAN	Q86917	29.65	172	113	6	33	199	77	245	7.00E-19	82.8
tr|C9JW47|C9JW47_HUMAN	Q9J5H4	30.99	142	94	3	53	191	32	172	2.00E-17	78.2
tr|C9JW47|C9JW47_HUMAN	P32229	38.89	90	54	1	92	180	1	90	3.00E-15	71.2
tr|C9JW47|C9JW47_HUMAN	F5HF62	34	150	87	5	35	179	19	161	6.00E-14	68.6
tr|C9JW47|C9JW47_HUMAN	P69333	34	150	87	5	35	179	19	161	9.00E-14	68.2
tr|C9JW47|C9JW47_HUMAN	P69332	34	150	87	5	35	179	19	161	9.00E-14	68.2
tr|C9JW47|C9JW47_HUMAN	Q9J5I0	30.07	153	101	4	51	199	20	170	5.00E-12	62.8
tr|C9JW47|C9JW47_HUMAN	Q89609	36.67	120	72	3	68	184	93	211	5.00E-11	60.1
tr|C9JW47|C9JW47_HUMAN	Q01035	30.85	188	122	5	15	201	7	187	5.00E-09	53.9
tr|C9JW47|C9JW47_HUMAN	Q98146	26.73	202	142	4	2	201	4	201	1.00E-08	53.1
tr|C9JW47|C9JW47_HUMA	Q9J529	28.08	146	92	5	53	191	22	161	3.00E	51.6

N										-08	
tr|C9JW47|C9JW47_HUMAN	F5HDK1	30.23	129	82	4	58	179	39	166	4.00E-07	48.5
tr|C9JW47|C9JW47_HUMAN	P09703	29.46	129	83	4	58	179	37	164	1.00E-06	47.4
tr|C9JW47|C9JW47_HUMAN	O12000	30.37	135	85	4	56	183	38	170	7.00E-04	38.9
tr|C9JW47|C9JW47_HUMAN	Q18LE5	23.23	99	71	2	50	145	25	121	0.005	36.2
tr|C9JW47|C9JW47_HUMAN	P16849	25	164	115	6	24	183	8	167	0.006	36.2
tr|C9JW47|C9JW47_HUMAN	Q83207	28.28	145	100	4	39	179	23	167	0.04	33.5
tr|C9JW47|C9JW47_HUMAN	Q6SW98	27.27	143	88	8	66	196	50	188	0.048	33.5
tr|C9JW47|C9JW47_HUMAN	P06501	28.36	67	39	1	123	189	76	133	5.5	26.6
tr|C9JW47|C9JW47_HUMAN	Q5I147	29.79	47	33	0	104	150	144	190	6.3	26.6
sp|P25025|CXCR2_HUMAN	Q08520	31.02	303	197	7	44	339	55	352	4.00E-45	160
sp|P25025|CXCR2_HUMAN	F5HF62	32.94	337	200	8	35	357	19	343	4.00E-42	152
sp|P25025|CXCR2_HUMAN	P69333	33.76	311	187	7	35	338	19	317	1.00E-41	150
sp|P25025|CXCR2_HUMAN	P69332	33.76	311	187	7	35	338	19	317	1.00E-41	150
sp|P25025|CXCR2_HUMAN	Q86917	29.57	301	199	9	33	328	77	369	9.00E-39	143
sp|P25025|CXCR2_HUMA	Q89609	35.23	264	160	7	68	325	93	351	8.00E	135

N										-36	
sp|P25025|CXCR2_HUMAN	P32229	29.02	255	170	6	92	339	1	251	2.00E-32	123
sp|P25025|CXCR2_HUMAN	Q01035	30.87	311	205	6	15	323	7	309	1.00E-31	122
sp|P25025|CXCR2_HUMAN	Q98146	29.63	324	222	4	2	323	4	323	2.00E-31	122
sp|P25025|CXCR2_HUMAN	Q9J5H4	25.55	274	186	9	53	317	32	296	5.00E-26	106
sp|P25025|CXCR2_HUMAN	Q9J5I0	28.73	275	179	7	51	319	20	283	4.00E-20	89
sp|P25025|CXCR2_HUMAN	Q9J529	24.91	281	188	10	53	319	22	293	1.00E-18	84.7
sp|P25025|CXCR2_HUMAN	F5HDK1	26.19	294	199	10	58	341	39	324	2.00E-17	81.6
sp|P25025|CXCR2_HUMAN	P09703	26.44	295	197	11	58	341	37	322	2.00E-17	81.6
sp|P25025|CXCR2_HUMAN	O12000	25.25	297	191	11	56	333	38	322	5.00E-10	59.7
sp|P25025|CXCR2_HUMAN	Q83207	23.78	307	211	10	39	329	23	322	6.00E-10	59.3
sp|P25025|CXCR2_HUMAN	P16849	22.47	316	217	13	24	321	8	313	1.00E-09	58.5
sp|P25025|CXCR2_HUMAN	Q6SW98	24.08	299	199	14	40	321	26	313	1.00E-09	58.2
sp|P25025|CXCR2_HUMAN	P52380	21.54	260	195	5	68	323	49	303	2.00E-05	45.4
sp|P25025|CXCR2_HUMAN	Q66673	28.12	96	69	0	231	326	230	325	4.00E-05	44.3
sp|P25025|CXCR2_HUMA	P52381	20.16	253	199	3	71	322	69	319	0.002	38.9

N											
sp|P25025|CXCR2_HUMAN	Q18LE5	23.23	99	71	2	50	145	25	121	0.007	37.4
sp|P25025|CXCR2_HUMAN	P06501	28.36	67	39	1	123	189	76	133	7.8	27.3
sp|P49682|CXCR3_HUMAN	F5HF62	30.55	347	216	5	26	362	13	344	1.00E-44	159
sp|P49682|CXCR3_HUMAN	P69333	31.69	325	203	5	26	349	13	319	1.00E-43	156
sp|P49682|CXCR3_HUMAN	P69332	31.69	325	203	5	26	349	13	319	1.00E-43	156
sp|P49682|CXCR3_HUMAN	Q89609	34.01	297	187	5	40	330	59	352	5.00E-38	141
sp|P49682|CXCR3_HUMAN	Q08520	26.64	289	184	9	58	329	64	341	5.00E-35	132
sp|P49682|CXCR3_HUMAN	Q86917	29.15	295	190	9	56	343	94	376	7.00E-30	118
sp|P49682|CXCR3_HUMAN	P32229	25.2	250	160	8	97	329	1	240	2.00E-27	109
sp|P49682|CXCR3_HUMAN	F5HDK1	27.18	298	195	5	73	353	53	345	2.00E-24	102
sp|P49682|CXCR3_HUMAN	P09703	27.18	298	195	5	73	353	51	343	7.00E-24	100
sp|P49682|CXCR3_HUMAN	Q9J5H4	24.47	282	194	8	50	321	24	296	3.00E-23	98.2
sp|P49682|CXCR3_HUMAN	Q9J529	25.37	272	187	8	62	322	26	292	1.00E-20	90.1
sp|P49682|CXCR3_HUMAN	Q98146	25.71	319	214	4	25	333	24	329	7.00E-17	80.1
sp|P49682|CXCR3_HUMA	Q01035	24.52	314	220	8	21	329	10	311	1.00E	79

N										-16	
sp|P49682|CXCR3_HUMAN	Q83207	25.22	345	234	9	28	353	8	347	3.00E-16	78.6
sp|P49682|CXCR3_HUMAN	Q9J5I0	25.82	306	203	11	59	353	23	315	1.00E-15	76.3
sp|P49682|CXCR3_HUMAN	P16849	23.9	318	202	12	34	329	18	317	6.00E-12	65.9
sp|P49682|CXCR3_HUMAN	O12000	26.41	284	181	13	64	328	41	315	4.00E-11	63.2
sp|P49682|CXCR3_HUMAN	Q6SW98	22.78	281	199	8	62	329	42	317	2.00E-09	57.8
sp|P49682|CXCR3_HUMAN	Q18LE5	22.81	228	154	6	53	272	23	236	4.00E-06	47.4
sp|P49682|CXCR3_HUMAN	P52381	20.47	337	249	9	19	348	17	341	2.00E-05	45.4
sp|P49682|CXCR3_HUMAN	P52380	20.92	306	213	8	74	367	50	338	2.00E-05	45.1
sp|P49682|CXCR3_HUMAN	Q66673	21.15	279	202	8	22	294	23	289	0.001	39.7
sp|P49682|CXCR3_HUMAN	P24025	30	40	28	0	207	246	154	193	8.6	27.7
sp|P61073|CXCR4_HUMAN	F5HF62	30.36	336	220	4	20	348	15	343	3.00E-47	165
sp|P61073|CXCR4_HUMAN	Q89609	32.2	295	194	3	26	315	60	353	7.00E-46	162
sp|P61073|CXCR4_HUMAN	P69333	31.27	291	194	2	20	310	15	299	3.00E-45	160
sp|P61073|CXCR4_HUMAN	P69332	31.27	291	194	2	20	310	15	299	3.00E-45	160
sp|P61073|CXCR4_HUMA	Q08520	27.51	309	210	7	31	327	49	355	3.00E	144

N										-39	
sp|P61073|CXCR4_HUMAN	Q86917	30.31	287	194	5	41	324	94	377	4.00E-37	138
sp|P61073|CXCR4_HUMAN	P32229	26.67	255	177	5	82	327	1	254	4.00E-31	119
sp|P61073|CXCR4_HUMAN	Q9J5H4	27.18	309	196	8	10	305	4	296	4.00E-31	120
sp|P61073|CXCR4_HUMAN	Q9J5I0	29.68	310	189	9	44	343	23	313	4.00E-20	88.6
sp|P61073|CXCR4_HUMAN	P09703	24.16	269	195	5	48	310	37	302	1.00E-15	76.3
sp|P61073|CXCR4_HUMAN	F5HDK1	24.16	269	195	5	48	310	39	304	3.00E-15	75.5
sp|P61073|CXCR4_HUMAN	Q83207	25	348	240	8	14	347	9	349	1.00E-14	73.9
sp|P61073|CXCR4_HUMAN	Q9J529	22.89	284	193	11	47	314	26	299	3.00E-14	71.6
sp|P61073|CXCR4_HUMAN	O12000	24.82	278	194	5	46	310	38	313	3.00E-11	63.5
sp|P61073|CXCR4_HUMAN	Q01035	23.43	303	221	6	9	310	16	308	9.00E-10	58.5
sp|P61073|CXCR4_HUMAN	P16849	22.33	309	209	10	19	309	18	313	1.00E-09	58.5
sp|P61073|CXCR4_HUMAN	Q6SW98	22.02	277	189	8	46	309	51	313	4.00E-09	56.6
sp|P61073|CXCR4_HUMAN	Q98146	22.55	275	200	5	44	310	53	322	2.00E-07	51.6
sp|P61073|CXCR4_HUMAN	P52380	23.96	288	199	5	73	352	65	340	1.00E-06	48.5
sp|P61073|CXCR4_HUMA	P52381	22.78	281	199	9	41	310	46	319	2.00E	48.1

N										-06	
sp|P61073|CXCR4_HUMAN	Q18LE5	30.84	107	66	4	35	135	17	121	7.00E-05	43.5
sp|P61073|CXCR4_HUMAN	Q66673	19.15	282	209	8	7	279	19	290	0.011	36.6
sp|P61073|CXCR4_HUMAN	P21060	41.67	24	14	0	172	195	159	182	4	28.5
sp|P61073|CXCR4_HUMAN	P24762	41.67	24	14	0	172	195	159	182	4.9	28.1
sp|O00574|CXCR6_HUMAN	Q89609	33.58	265	161	6	49	300	90	352	7.00E-37	137
sp|O00574|CXCR6_HUMAN	Q08520	25.47	318	210	7	3	296	24	338	4.00E-34	130
sp|O00574|CXCR6_HUMAN	Q86917	28.88	277	182	8	37	302	97	369	7.00E-30	117
sp|O00574|CXCR6_HUMAN	F5HF62	27.54	305	196	6	3	298	13	301	1.00E-27	110
sp|O00574|CXCR6_HUMAN	P69333	27.21	305	197	6	3	298	13	301	3.00E-27	109
sp|O00574|CXCR6_HUMAN	P69332	27.21	305	197	6	3	298	13	301	3.00E-27	109
sp|O00574|CXCR6_HUMAN	P32229	25.73	241	160	5	75	298	1	239	5.00E-26	105
sp|O00574|CXCR6_HUMAN	F5HDK1	25.9	278	189	9	43	308	44	316	8.00E-19	85.5
sp|O00574|CXCR6_HUMAN	P09703	25.54	278	190	9	43	308	42	314	3.00E-18	84
sp|O00574|CXCR6_HUMAN	Q9J5H4	23.41	299	204	9	14	291	2	296	3.00E-16	77.8
sp|O00574|CXCR6_HUMA	Q9J529	24.63	272	183	9	39	292	25	292	2.00E	75.1

N										-15	
sp|O00574|CXCR6_HUMAN	Q9J5I0	23.97	267	185	9	37	292	23	282	1.00E-09	57.8
sp|O00574|CXCR6_HUMAN	P16849	24.65	284	175	12	40	295	41	313	1.00E-08	55.5
sp|O00574|CXCR6_HUMAN	Q83207	21.63	319	216	8	16	303	7	322	3.00E-08	54.3
sp|O00574|CXCR6_HUMAN	Q6SW98	24.03	283	177	12	40	295	42	313	2.00E-07	51.6
sp|O00574|CXCR6_HUMAN	Q01035	22.08	317	221	7	8	306	10	318	1.00E-06	48.5
sp|O00574|CXCR6_HUMAN	O12000	25	284	183	11	38	299	41	316	2.00E-06	48.5
sp|O00574|CXCR6_HUMAN	Q18LE5	23.71	291	182	11	67	333	64	338	1.00E-04	42.7
sp|O00574|CXCR6_HUMAN	P52381	21.78	326	211	12	2	304	23	327	0.002	38.9
sp|O00574|CXCR6_HUMAN	Q66673	22.61	261	179	9	5	253	23	272	0.23	32.3
sp|O00574|CXCR6_HUMAN	Q98146	20.07	284	203	7	40	306	56	332	1.2	30
sp|O00574|CXCR6_HUMAN	Q84424	41.03	39	22	1	167	205	224	261	1.2	30
sp|O00590|ACKR2_HUMAN	Q86917	34.04	282	179	5	43	323	91	366	3.00E-49	172
sp|O00590|ACKR2_HUMAN	Q89609	33.44	308	193	5	36	336	62	364	5.00E-41	150
sp|O00590|ACKR2_HUMAN	Q08520	29.63	297	190	7	46	330	59	348	3.00E-40	147
sp|O00590|ACKR2_HUMA	F5HF62	28.62	325	208	6	53	365	40	352	1.00E	145

N										-39	
sp|O00590|ACKR2_HUMAN	P69333	31.07	280	177	5	53	328	40	307	7.00E-39	143
sp|O00590|ACKR2_HUMAN	P69332	31.07	280	177	5	53	328	40	307	7.00E-39	143
sp|O00590|ACKR2_HUMAN	P32229	29.64	253	160	6	90	330	1	247	9.00E-34	127
sp|O00590|ACKR2_HUMAN	F5HDK1	24.44	270	196	5	55	321	41	305	1.00E-21	94
sp|O00590|ACKR2_HUMAN	P09703	24.44	270	196	5	55	321	39	303	3.00E-21	92.8
sp|O00590|ACKR2_HUMAN	Q9J5H4	23.08	286	190	11	44	314	25	295	1.00E-20	90.9
sp|O00590|ACKR2_HUMAN	Q9J5I0	27	237	161	7	45	275	16	246	5.00E-17	80.5
sp|O00590|ACKR2_HUMAN	Q9J529	24.29	247	171	8	81	316	51	292	1.00E-15	76.3
sp|O00590|ACKR2_HUMAN	P16849	24.12	340	223	10	55	373	41	366	5.00E-15	75.5
sp|O00590|ACKR2_HUMAN	Q6SW98	25.43	291	182	9	55	324	42	318	1.00E-13	71.2
sp|O00590|ACKR2_HUMAN	Q98146	25.74	272	195	4	54	321	55	323	2.00E-13	69.7
sp|O00590|ACKR2_HUMAN	Q83207	22.68	291	206	7	54	332	41	324	3.00E-11	63.9
sp|O00590|ACKR2_HUMAN	O12000	20.48	249	182	7	83	321	72	314	4.00E-07	50.8
sp|O00590|ACKR2_HUMAN	Q18LE5	29.35	92	64	1	53	143	30	121	3.00E-05	44.7
sp|O00590|ACKR2_HUMA	Q01035	22.02	327	231	9	14	330	6	318	7.00E	43.5

N										-05	
sp|O00590|ACKR2_HUMAN	P52381	19.23	156	123	2	167	320	165	319	0.021	35.8
sp|O00590|ACKR2_HUMAN	P52380	18.92	259	204	5	69	325	53	307	0.042	35
sp|O00590|ACKR2_HUMAN	Q66673	22.18	284	197	8	2	277	10	277	3.7	28.9
sp|O00590|ACKR2_HUMAN	Q5UNV7	41.18	34	17	1	116	146	88	121	4.3	28.5
tr|V9GYB7|V9GYB7_HUMAN	Q86917	37.5	192	114	4	43	233	91	277	7.00E-35	129
tr|V9GYB7|V9GYB7_HUMAN	Q08520	31.98	197	118	6	46	233	59	248	2.00E-25	102
tr|V9GYB7|V9GYB7_HUMAN	P32229	32.89	152	87	5	91	233	2	147	5.00E-19	82.8
tr|V9GYB7|V9GYB7_HUMAN	Q89609	32.32	198	130	3	36	230	62	258	2.00E-18	82.4
tr|V9GYB7|V9GYB7_HUMAN	Q9J5H4	23.62	199	139	6	44	233	25	219	3.00E-15	73.2
tr|V9GYB7|V9GYB7_HUMAN	F5HF62	26.63	184	120	4	53	233	40	211	4.00E-15	72.4
tr|V9GYB7|V9GYB7_HUMAN	P69333	26.63	184	120	4	53	233	40	211	5.00E-15	72.4
tr|V9GYB7|V9GYB7_HUMAN	P69332	26.63	184	120	4	53	233	40	211	5.00E-15	72.4
tr|V9GYB7|V9GYB7_HUMAN	Q9J5I0	27.37	190	133	4	45	230	16	204	1.00E-13	67.8
tr|V9GYB7|V9GYB7_HUMAN	F5HDK1	24.73	182	129	5	55	233	41	217	2.00E-13	68.2
tr|V9GYB7|V9GYB7_HUM	P09703	24.73	182	129	5	55	233	39	215	2.00E	67.8

AN										-13	
tr|V9GYB7|V9GYB7_HUMAN	Q9J529	23.9	159	112	4	81	233	51	206	2.00E-07	50.1
tr|V9GYB7|V9GYB7_HUMAN	P16849	28.76	153	100	5	55	204	41	187	1.00E-06	48.1
tr|V9GYB7|V9GYB7_HUMAN	Q18LE5	27.27	99	71	1	46	143	23	121	2.00E-05	44.3
tr|V9GYB7|V9GYB7_HUMAN	Q6SW98	26.15	195	115	7	55	231	42	225	2.00E-05	44.3
tr|V9GYB7|V9GYB7_HUMAN	Q83207	21.34	164	123	2	26	183	9	172	5.00E-04	39.7
tr|V9GYB7|V9GYB7_HUMAN	Q98146	24.86	181	130	3	54	231	55	232	0.002	38.1
tr|V9GYB7|V9GYB7_HUMAN	O12000	20.54	112	86	2	83	192	72	182	0.067	33.1
tr|V9GYB7|V9GYB7_HUMAN	Q5UNV7	41.18	34	17	1	116	146	88	121	1.4	28.9
tr|V9GYB7|V9GYB7_HUMAN	P13897	34.88	43	20	2	171	205	1073	1115	3.7	28.1
tr|V9GYB7|V9GYB7_HUMAN	Q8JJX0	33.87	62	32	3	153	205	1132	1193	7.9	26.9
tr|V9GYB7|V9GYB7_HUMAN	Q8QL52	33.87	62	32	3	153	205	1133	1194	7.9	26.9
tr|V9GYM0|V9GYM0_HUMAN	Q08520	34.18	79	51	1	46	124	59	136	3.00E-09	53.1
tr|V9GYM0|V9GYM0_HUMAN	Q86917	40.96	83	44	3	43	124	91	169	3.00E-09	53.1
tr|V9GYM0|V9GYM0_HUMAN	Q89609	41.86	86	48	1	36	119	62	147	1.00E-06	45.4
tr|V9GYM0|V9GYM0_HU	Q9J5H4	28.57	84	57	2	44	125	25	107	1.00E	45.4

MAN										-06	
tr|V9GYM0|V9GYM0_HUMAN	Q9J5I0	28.92	83	56	2	45	125	16	97	5.00E-05	40.4
tr|V9GYM0|V9GYM0_HUMAN	F5HF62	30.67	75	46	2	53	124	40	111	1.00E-04	39.3
tr|V9GYM0|V9GYM0_HUMAN	P69333	30.67	75	46	2	53	124	40	111	2.00E-04	38.9
tr|V9GYM0|V9GYM0_HUMAN	P69332	30.67	75	46	2	53	124	40	111	2.00E-04	38.9
tr|V9GYM0|V9GYM0_HUMAN	Q18LE5	32.43	74	49	1	53	125	30	103	6.00E-04	37.4
tr|V9GYM0|V9GYM0_HUMAN	P32229	41.18	34	20	0	91	124	2	35	0.013	33.5
tr|V9GYM0|V9GYM0_HUMAN	P16849	35.21	71	45	1	55	124	41	111	0.076	31.2
tr|V9GYM0|V9GYM0_HUMAN	F5HDK1	33.33	48	31	1	55	101	41	88	0.1	30.8
tr|V9GYM0|V9GYM0_HUMAN	P09703	33.33	48	31	1	55	101	39	86	0.13	30.8
tr|V9GYM0|V9GYM0_HUMAN	Q9J529	36.17	47	24	2	81	123	51	95	0.18	30
tr|V9GYM0|V9GYM0_HUMAN	Q6SW98	33.8	71	46	1	55	124	42	112	0.66	28.5
tr|V9GYM0|V9GYM0_HUMAN	Q83207	27.4	73	51	1	54	124	41	113	5.8	25.8
tr|V9GYX0|V9GYX0_HUMAN	Q86917	39.06	128	73	3	43	169	91	214	1.00E-20	87
tr|V9GYX0|V9GYX0_HUMAN	Q08520	35.43	127	75	3	46	169	59	181	4.00E-19	82.8
tr|V9GYX0|V9GYX0_HUM	Q89609	36.03	136	85	1	36	169	62	197	2.00E	67

AN										-13	
tr|V9GYX0|V9GYX0_HUMAN	P32229	39.02	82	44	2	91	169	2	80	1.00E-12	63.5
tr|V9GYX0|V9GYX0_HUMAN	Q9J5H4	30.7	114	72	3	44	155	25	133	1.00E-12	63.9
tr|V9GYX0|V9GYX0_HUMAN	Q9J5I0	27.05	122	86	2	45	164	16	136	3.00E-10	56.6
tr|V9GYX0|V9GYX0_HUMAN	F5HF62	29.17	120	75	3	53	169	40	152	2.00E-09	55.1
tr|V9GYX0|V9GYX0_HUMAN	P69333	29.17	120	75	3	53	169	40	152	2.00E-09	54.7
tr|V9GYX0|V9GYX0_HUMAN	P69332	29.17	120	75	3	53	169	40	152	2.00E-09	54.7
tr|V9GYX0|V9GYX0_HUMAN	F5HDK1	25.64	117	85	2	55	169	41	157	6.00E-07	47.4
tr|V9GYX0|V9GYX0_HUMAN	P09703	25.64	117	85	2	55	169	39	155	7.00E-07	47
tr|V9GYX0|V9GYX0_HUMAN	Q9J529	27.96	93	61	2	81	169	51	141	4.00E-06	44.7
tr|V9GYX0|V9GYX0_HUMAN	Q18LE5	29.35	92	64	1	53	143	30	121	7.00E-06	44.3
tr|V9GYX0|V9GYX0_HUMAN	P16849	29.73	111	76	2	55	164	41	150	1.00E-04	40.4
tr|V9GYX0|V9GYX0_HUMAN	Q6SW98	28.83	111	77	2	55	164	42	151	0.001	37.7
tr|V9GYX0|V9GYX0_HUMAN	Q98146	25	96	72	0	54	149	55	150	0.005	35.8
tr|V9GYX0|V9GYX0_HUMAN	Q83207	22.88	118	89	1	54	169	41	158	0.015	34.3
tr|V9GYX0|V9GYX0_HUM	O12000	20.69	87	68	1	83	169	72	157	0.87	28.9

AN											
tr|V9GYX0|V9GYX0_HUMAN	Q5UNV7	41.18	34	17	1	116	146	88	121	1	28.9
tr|V9GZ70|V9GZ70_HUMAN	Q08520	35.05	97	62	1	46	142	59	154	5.00E-14	67.8
tr|V9GZ70|V9GZ70_HUMAN	Q86917	40.59	101	55	3	43	142	91	187	9.00E-14	67
tr|V9GZ70|V9GZ70_HUMAN	Q9J5H4	29.7	101	68	2	44	142	25	124	6.00E-11	58.5
tr|V9GZ70|V9GZ70_HUMAN	Q89609	37.61	109	66	1	36	142	62	170	3.00E-09	53.9
tr|V9GZ70|V9GZ70_HUMAN	Q9J5I0	30	100	67	2	45	142	16	114	3.00E-09	53.5
tr|V9GZ70|V9GZ70_HUMAN	P32229	40.38	52	31	0	91	142	2	53	1.00E-07	48.1
tr|V9GZ70|V9GZ70_HUMAN	F5HF62	29.03	93	60	2	53	142	40	129	2.00E-07	47.8
tr|V9GZ70|V9GZ70_HUMAN	P69333	29.03	93	60	2	53	142	40	129	3.00E-07	47.4
tr|V9GZ70|V9GZ70_HUMAN	P69332	29.03	93	60	2	53	142	40	129	3.00E-07	47.4
tr|V9GZ70|V9GZ70_HUMAN	Q18LE5	29.67	91	63	1	53	142	30	120	9.00E-06	43.1
tr|V9GZ70|V9GZ70_HUMAN	Q9J529	34.85	66	37	2	81	142	51	114	4.00E-04	38.1
tr|V9GZ70|V9GZ70_HUMAN	F5HDK1	25.56	90	65	2	55	142	41	130	0.003	35.8
tr|V9GZ70|V9GZ70_HUMAN	P09703	25.56	90	65	2	55	142	39	128	0.004	35.4
tr|V9GZ70|V9GZ70_HUMA	P16849	30.34	89	61	1	55	142	41	129	0.076	31.6

N											
tr|V9GZ70|V9GZ70_HUMAN	Q98146	24.72	89	67	0	54	142	55	143	0.24	30.4
tr|V9GZ70|V9GZ70_HUMAN	Q6SW98	29.21	89	62	1	55	142	42	130	0.54	29.3
tr|V9GZ70|V9GZ70_HUMAN	Q83207	24.18	91	67	1	54	142	41	131	1.5	27.7
tr|C9JNR0|C9JNR0_HUMAN	P25212	31.7	224	138	4	8	229	6	216	6.00E-34	125
tr|C9JNR0|C9JNR0_HUMAN	O57261	31.7	224	138	4	8	229	6	216	7.00E-34	125
tr|C9JNR0|C9JNR0_HUMAN	P21116	31.7	224	138	4	8	229	6	216	7.00E-34	125
tr|C9JNR0|C9JNR0_HUMAN	Q04523	31.4	207	127	4	25	229	23	216	2.00E-30	116
tr|C9JNR0|C9JNR0_HUMAN	P33795	24.71	174	112	7	43	211	69	228	3.00E-04	40.4
tr|C9JNR0|C9JNR0_HUMAN	P23998	21.81	188	130	7	27	211	52	225	0.02	34.7
tr|C9JNR0|C9JNR0_HUMAN	P21077	21.81	188	130	7	27	211	54	227	0.023	34.7
tr|C9JNR0|C9JNR0_HUMAN	P25213	21.81	188	130	7	27	211	52	225	0.029	34.3
tr|C9JNR0|C9JNR0_HUMAN	Q37893	48.28	29	15	0	6	34	413	441	0.76	30
tr|C9JNR0|C9JNR0_HUMAN	P03218	25.88	85	44	2	159	228	71	151	1.1	29.3
tr|C9JNR0|C9JNR0_HUMAN	P20747	33.33	42	23	1	188	229	197	233	1.5	28.9
tr|C9JNR0|C9JNR0_HUMA	P31792	39.22	51	27	2	154	204	181	227	2.4	28.5

N											
tr|C9JNR0|C9JNR0_HUMAN	P0C788	31.43	35	23	1	192	226	94	127	2.7	28.1
tr|C9JNR0|C9JNR0_HUMAN	Q06998	31.11	45	27	1	39	83	414	454	4.1	27.7
tr|C9JNR0|C9JNR0_HUMAN	P0C9T7	22.31	121	71	3	53	157	15	128	4.9	27.3
tr|C9JNR0|C9JNR0_HUMAN	P89466	44.44	36	20	0	1	36	47	82	5.4	27.3
tr|C9JNR0|C9JNR0_HUMAN	P33840	25.37	67	50	0	125	191	1	67	8.7	25.4
sp|P27930|IL1R2_HUMAN	P25212	30.65	323	203	8	8	327	6	310	2.00E-39	144
sp|P27930|IL1R2_HUMAN	O57261	30.34	323	204	8	8	327	6	310	2.00E-39	144
sp|P27930|IL1R2_HUMAN	P21116	30.28	327	199	9	8	327	6	310	2.00E-38	142
sp|P27930|IL1R2_HUMAN	Q04523	29.74	306	194	8	25	327	23	310	7.00E-36	135
sp|P27930|IL1R2_HUMAN	P33795	24.68	308	198	12	43	341	69	351	2.00E-07	52
sp|P27930|IL1R2_HUMAN	P23998	23.29	322	215	12	27	341	52	348	4.00E-07	50.8
sp|P27930|IL1R2_HUMAN	P25213	23.29	322	215	12	27	341	52	348	5.00E-07	50.4
sp|P27930|IL1R2_HUMAN	P21077	23.29	322	215	12	27	341	54	350	6.00E-07	50.1
sp|P27930|IL1R2_HUMAN	P20747	31.34	67	33	3	188	251	197	253	0.92	30.4
sp|P27930|IL1R2_HUMAN	P52463	31.88	69	32	2	232	291	182	244	1.7	30
sp|P27930|IL1R2_HUMAN	Q37893	48.28	29	15	0	6	34	413	441	1.9	30

sp|P27930|IL1R2_HUMAN	P03218	25.88	85	44	2	159	228	71	151	2.4	29.3
sp|P27930|IL1R2_HUMAN	P33840	25.37	67	50	0	125	191	1	67	2.6	27.7
sp|P27930|IL1R2_HUMAN	P0C788	31.43	35	23	1	192	226	94	127	3.9	28.5
sp|P27930|IL1R2_HUMAN	P31792	39.22	51	27	2	154	204	181	227	4.2	28.9
sp|P27930|IL1R2_HUMAN	P0C9U3	28.41	88	52	4	263	342	66	150	5.5	28.5
sp|P27930|IL1R2_HUMAN	P0C9U5	27.27	88	53	4	263	342	66	150	6	28.1
sp|P27930|IL1R2_HUMAN	P0C9U1	26.14	88	54	3	263	342	66	150	7.3	28.1
sp|P27930|IL1R2_HUMAN	Q9QJ33	30.43	69	33	2	232	291	182	244	7.9	27.7
sp|P27930|IL1R2_HUMAN	P84400	24.72	178	109	6	196	367	200	358	8.7	27.7
sp|P27930|IL1R2_HUMAN	P28959	24.72	178	109	6	196	367	200	358	8.7	27.7
sp|P27930|IL1R2_HUMAN	P89466	44.44	36	20	0	1	36	47	82	8.7	27.7
sp|P27930|IL1R2_HUMAN	Q06998	26.15	65	43	2	39	102	414	474	9.2	27.7
sp|P22301|IL10_HUMAN	P68677	75.98	179	42	1	1	178	1	179	1.00E-94	275
sp|P22301|IL10_HUMAN	P68678	75.98	179	42	1	1	178	1	179	1.00E-94	275
sp|P22301|IL10_HUMAN	P0CAP9	82.46	171	21	3	7	177	9	170	1.00E-79	237
sp|P22301|IL10_HUMAN	P03180	82.46	171	21	3	7	177	9	170	1.00E-79	237
sp|P22301|IL10_HUMAN	P0C6Z6	82.46	171	21	3	7	177	9	170	1.00E-79	237
sp|P22301|IL10_HUMAN	F5HC71	26.59	173	120	5	1	172	6	172	1.00E-13	65.1
sp|P22301|IL10_HUMAN	P17150	26.16	172	121	4	1	172	6	171	2.00E-13	64.7
sp|P22301|IL10_HUMAN	Q197A4	22.22	90	67	2	62	149	16	104	0.45	30
sp|P22301|IL10_HUMAN	P03352	22.97	74	54	1	16	86	25	98	3.1	27.3
sp|P22301|IL10_HUMAN	P23425	22.97	74	54	1	16	86	25	98	3.2	27.3

sp|P22301|IL10_HUMAN	P35955	22.97	74	54	1	16	86	25	98	3.3	27.3
sp|P22301|IL10_HUMAN	P23424	22.97	74	54	1	16	86	25	98	3.3	27.3
sp|P22301|IL10_HUMAN	Q196T9	25	80	36	2	78	147	853	918	6	26.6
sp|P22301|IL10_HUMAN	Q8JJE2	33.33	33	22	0	145	177	257	289	6.4	26.6
sp|P22301|IL10_HUMAN	P0C6T6	40.74	27	15	1	142	168	2848	2873	7.6	26.6
sp|P22301|IL10_HUMAN	P0C6W5	40.74	27	15	1	142	168	2848	2873	7.6	26.6
sp|P22301|IL10_HUMAN	P16472	42.31	26	15	0	146	171	199	224	9.2	25.8
sp|Q16552|IL17_HUMAN	P24916	71.22	139	37	2	16	154	15	150	3.00E-67	204
sp|Q16552|IL17_HUMAN	O40633	70.78	154	41	3	1	154	1	150	2.00E-66	201
sp|Q16552|IL17_HUMAN	F5HAW0	30.61	49	25	1	38	77	49	97	5	26.6
sp|Q16552|IL17_HUMAN	P30318	32.26	31	21	0	67	97	823	853	5.8	26.6
sp|Q96PD4|IL17F_HUMAN	P24916	53.64	110	51	0	54	163	42	151	6.00E-35	121
sp|Q96PD4|IL17F_HUMAN	O40633	52.73	110	52	0	54	163	42	151	2.00E-34	120
sp|Q96PD4|IL17F_HUMAN	P31332	37.5	48	23	2	109	156	1224	1264	4.7	26.9
sp|Q96PD4|IL17F_HUMAN	B2BNE1	32.69	52	34	1	36	86	221	272	9	26.2
sp|Q96PD4|IL17F_HUMAN	P0C9P1	23.44	64	46	1	53	116	55	115	9.7	25.8
sp|Q96PD4|IL17F_HUMAN	P0C9N7	23.44	64	46	1	53	116	55	115	9.7	25.8

Supplementary Table S5. List of 151 viral proteins share similarity higher then 30% with 27 human immune related proteins

Entry	Gene names	Organism	Taxonomic lineage (FAMILY)
P0C788	K14	Human herpesvirus 8 type P (isolate GK18) (HHV-8) (Kaposi's sarcoma-associated herpesvirus)	Herpesviridae
Q9QJ17	U85	Human herpesvirus 6B (strain Z29) (HHV-6 variant B) (Human B lymphotropic virus)	Herpesviridae
P35934	S2	Broadhaven virus (BRD)	Reoviridae
Q5GA87	M	Taro vein chlorosis virus (TAVCV)	Rhabdoviridae
P12439	HA	Influenza A virus (strain A/Mink/Sweden/1984 H10N4)	Orthomyxoviridae
Q8UYT3	ORF2b	Tomato aspermy virus (TAV)	Bromoviridae
P36693	2	Enterobacteria phage P21 (Bacteriophage 21) (Bacteriophage P21)	Siphoviridae (phages with long non-contractile tails)
P27330	ORF2	Lily symptomless virus (LSV)	Betaflexiviridae
Q04995	env	Feline immunodeficiency virus (strain UK8) (FIV)	Retroviridae
Q05312	env	Feline immunodeficiency virus (isolate Wo) (FIV)	Retroviridae
P19030	env	Feline immunodeficiency virus (strain San Diego) (FIV)	Retroviridae
P16090	env	Feline immunodeficiency virus (isolate Petaluma) (FIV)	Retroviridae
P33807	HA A56R J9R	Variola virus (isolate Human/India/Ind3/1967) (VARV) (Smallpox virus)	Poxviridae
Q03804	env	Feline immunodeficiency virus (strain UT-113) (FIV)	Retroviridae
Q02282	env	Feline immunodeficiency virus (isolate TM2) (FIV)	Retroviridae
Q04993	env	Feline immunodeficiency virus (strain UK2) (FIV)	Retroviridae
Q89738	U85 EDLF1	Human herpesvirus 6A (strain Uganda-1102) (HHV-6 variant A) (Human B lymphotropic virus)	Herpesviridae
P03253	L3	Human adenovirus C serotype 5 (HAdV-5) (Human adenovirus 5)	Adenoviridae

P03252	L3	Human adenovirus C serotype 2 (HAdV-2) (Human adenovirus 2)	Adenoviridae
P11209	F	Human respiratory syncytial virus A (strain RSS-2)	Paramyxoviridae
P15630	ORF II	Soybean chlorotic mottle virus	Caulimoviridae
Q00996	15	Saimiriine herpesvirus 2 (strain 11) (SaHV-2) (Herpesvirus saimiri)	Herpesviridae
O71188	1a	Grapevine leafroll-associated virus 3 (isolate United States/NY1) (GLRaV-3) (Grapevine leafroll-associated closterovirus (isolate 109))	Closteroviridae
O71189	1a-1b	Grapevine leafroll-associated virus 3 (isolate United States/NY1) (GLRaV-3) (Grapevine leafroll-associated closterovirus (isolate 109))	Closteroviridae
Q86117	ORF1	Rabbit hemorrhagic disease virus (strain SD) (Ra/LV/RHDV/SD/1989/FR) (RHDV-SD)	Caliciviridae
Q86119	ORF1	Rabbit hemorrhagic disease virus (strain AST89) (Ra/LV/RHDV/AST89/1989/SP) (RHDV-AST89)	Caliciviridae
P69514		Cucumber green mottle mosaic virus (strain watermelon SH) (CGMMV)	Virgaviridae
P27410	ORF1	Rabbit hemorrhagic disease virus (strain Rabbit/Germany/FRG/1989) (Ra/LV/RHDV/GH/1989/GE) (RHDV-FRG)	Caliciviridae
P27411	ORF1	Rabbit hemorrhagic disease virus (strain V-351) (Ra/LV/RHDV/V351/1991/CK) (RHDV-V351)	Caliciviridae
Q89273	ORF1	Rabbit hemorrhagic disease virus (strain BS89) (Ra/LV/RHDV/BS89/1989/IT) (RHDV-BS89)	Caliciviridae
P12911	S	Chimpanzee hepatitis B virus (isolate United Kingdom/LSH/1988) (HBVcpz)	Hepadnaviridae
Q775J3	gH ORF37	Varicella-zoster virus (strain Oka vaccine) (HHV-3) (Human herpesvirus 3)	Herpesviridae
P09260	gH ORF37	Varicella-zoster virus (strain Dumas) (HHV-3) (Human	Herpesviridae

		herpesvirus 3)	
P10190	UL6	Human herpesvirus 1 (strain 17) (HHV-1) (Human herpes simplex virus 1)	Herpesviridae
P18557	BA71V-013 BA71V-014 V82L X'82	African swine fever virus (strain Badajoz 1971 Vero-adapted) (Ba71V) (ASFV)	Asfarviridae
P0C9H4	War-011	African swine fever virus (isolate Warthog/Namibia/Wart80/1980) (ASFV)	Asfarviridae
P0C9H6	Pret-013	African swine fever virus (isolate Tick/South Africa/Pretoriuskop Pr4/1996) (ASFV)	Asfarviridae
P26705	LIS121-1	African swine fever virus (isolate Portugal/Lis 57/1957) (ASFV)	Asfarviridae
Q9E6Q9	MDV019	Gallid herpesvirus 2 (strain Chicken/Md5/ATCC VR-987) (GaHV-2) (Marek's disease herpesvirus type 1)	Herpesviridae
P12726	alt	Enterobacteria phage T4 (Bacteriophage T4)	Myoviridae (phages with contractile tails)
P04048	V-KIT	Feline sarcoma virus (strain Hardy-Zuckerman 4)	Retroviridae
P0C9H5	Mal-010	African swine fever virus (isolate Tick/Malawi/Lil 20-1/1983) (ASFV)	Asfarviridae
P26709	LIS137	African swine fever virus (isolate Portugal/Lis 57/1957) (ASFV)	Asfarviridae
Q6DN67	L	Citrus psorosis virus (isolate Spain/P-121) (CPsV) (Citrus ringspot virus)	Ophioviridae
P14371	FPV239	Fowlpox virus (strain NVSL) (FPV)	Poxviridae
P14370	FPV008; FPV253	Fowlpox virus (strain NVSL) (FPV)	Poxviridae
O89335	Pret-069	African swine fever virus (isolate Tick/South Africa/Pretoriuskop Pr4/1996) (ASFV)	Asfarviridae
Q65150	Ba71V-057 EP153R	African swine fever virus (strain Badajoz 1971 Vero-adapted) (Ba71V) (ASFV)	Asfarviridae
P24765	VACWR165 A40R	Vaccinia virus (strain Western Reserve) (VACV) (Vaccinia virus (strain WR))	Poxviridae
P0CA63	Ken-069	African swine fever virus (isolate Pig/Kenya/KEN-50/1950) (ASFV)	Asfarviridae

P0CA65	War-067	African swine fever virus (isolate Warthog/Namibia/Wart80/1980) (ASFV)	Asfarviridae
Q65652	ORF1	Blueberry scorch virus (BBScV)	Betaflexiviridae
P25328		Saccharomyces 20S RNA narnavirus (ScNV-20S)	Narnaviridae
Q89609	E1	Equine herpesvirus 2 (strain 86/87) (EHV-2)	Herpesviridae
F5HF62	US28	Human cytomegalovirus (strain Merlin) (HHV-5) (Human herpesvirus 5)	Herpesviridae
P69333	US28	Human cytomegalovirus (strain Towne) (HHV-5) (Human herpesvirus 5)	Herpesviridae
P69332	US28	Human cytomegalovirus (strain AD169) (HHV-5) (Human herpesvirus 5)	Herpesviridae
Q86917	Q2/3L	Sheeppox virus (strain KS-1) (SPPV) (Capripoxvirus (strain KS-1))	Poxviridae
Q9J5I0	FPV021	Fowlpox virus (strain NVSL) (FPV)	Poxviridae
P19560	gag-pol	Bovine immunodeficiency virus (strain R29) (BIV) (Bovine immunodeficiency-like virus)	Retroviridae
Q6R7D6	ORF93	Ostreid herpesvirus 1 (isolate France) (OsHV-1) (Pacific oyster herpesvirus)	Malacoherpesviridae
Q08520	K2R	Swinepox virus (strain Kasza) (SWPV)	Poxviridae
P32229	C3L	Swinepox virus (strain Kasza) (SWPV)	Poxviridae
P42540		Acholeplasma phage L2 (Bacteriophage L2)	Plasmaviridae
Q9J5H4	FPV027	Fowlpox virus (strain NVSL) (FPV)	Poxviridae
F5HDK1	US27	Human cytomegalovirus (strain Merlin) (HHV-5) (Human herpesvirus 5)	Herpesviridae
P09703	US27	Human cytomegalovirus (strain AD169) (HHV-5) (Human herpesvirus 5)	Herpesviridae
Q9J529	FPV206	Fowlpox virus (strain NVSL) (FPV)	Poxviridae
Q69422		Hepatitis GB virus B (GBV-B) (GB virus B)	Flaviviridae
P05842		Bombyx mori densovirus (BmDNV) (Bombyx densonucleosis virus)	Parvoviridae

Q8B912	rep 1a-1b	Porcine reproductive and respiratory syndrome virus (strain HB-1) (PRRSV)	Arteriviridae
Q9YN02	rep 1a-1b	Porcine reproductive and respiratory syndrome virus (strain 16244B) (PRRSV)	Arteriviridae
Q9WJB2	rep 1a-1b	Porcine reproductive and respiratory syndrome virus (strain VR-2332) (PRRSV)	Arteriviridae
Q6UDM4	UL54	Psittacid herpesvirus 1 (isolate Amazon parrot/-/97-0001/1997) (PsHV-1) (Pacheco's disease virus)	Herpesviridae
A4ZUD1	ORF346	Acidianus bottle-shaped virus (isolate Italy/Pozzuoli) (ABV)	Ampullaviridae
P16838	UL129	Human cytomegalovirus (strain AD169) (HHV-5) (Human herpesvirus 5)	Herpesviridae
Q8V5U0	VCATH 57	Heliothis zea nuclear polyhedrosis virus (HzSNPV) (Helicoverpa zea single nucleocapsid nuclear polyhedrosis virus)	Baculoviridae
Q98146	ORF74	Human herpesvirus 8 type P (isolate GK18) (HHV-8) (Kaposi's sarcoma-associated herpesvirus)	Herpesviridae
P22958	ORF1	Tobacco necrosis virus (strain A) (TNV-A)	Tombusviridae
Q1A243	env	Simian immunodeficiency virus (isolate EK505) (SIV-cpz) (Chimpanzee immunodeficiency virus)	Retroviridae
Q88899		Tobacco rattle virus (isolate PpK20) (TRV)	Virgaviridae
Q8JVC1	p2	Penicillium chrysogenum virus (isolate Caston/2003) (PcV)	Chrysoviridae
Q2Y0E7	S4	Aedes pseudoscutellaris reovirus (isolate France) (ApRV)	Reoviridae
Q65900		Coxsackievirus A16 (strain G-10)	Picornaviridae
Q66478		Human enterovirus 71 (strain BrCr) (Ev 71)	Picornaviridae
Q66479		Human enterovirus 71 (strain 7423/MS/87) (Ev 71)	Picornaviridae
P14360	FPV240	Fowlpox virus (strain NVSL) (FPV)	Poxviridae
Q66431	L	Dugbe virus (isolate ArD44313) (DUGV)	Bunyaviridae
P0C6Y0	rep 1a-1b	Murine coronavirus (strain JHM) (MHV-JHM) (Murine hepatitis virus)	Coronaviridae
P0C6V1	1a	Murine coronavirus (strain JHM) (MHV-JHM) (Murine hepatitis virus)	Coronaviridae

Q69140	EBNA6 BERF3-BERF4	Epstein-Barr virus (strain AG876) (HHV-4) (Human herpesvirus 4)	Herpesviridae
P36216	CVIJIM	Paramecium bursaria Chlorella virus IL3A (PBCV-IL3A)	Phycodnaviridae
P27315	GP	Prospect Hill virus (PHV)	Bunyaviridae
Q83887	GP	New York virus (NYV)	Bunyaviridae
O10438	L1	Murine adenovirus A serotype 1 (MAdV-1) (Murine adenovirus 1)	Adenoviridae
P41423		Autographa californica nuclear polyhedrosis virus (AcMNPV)	Baculoviridae
Q88898		Tobacco rattle virus (isolate PpK20) (TRV)	Virgaviridae
Q91FV8	IIV6-213R	Invertebrate iridescent virus 6 (IIV-6) (Chilo iridescent virus)	Iridoviridae
Q05221	15	Mycobacterium phage L5 (Mycobacteriophage L5)	Siphoviridae (phages with long non-contractile tails)
P0C6V2	1a	Porcine transmissible gastroenteritis coronavirus (strain Purdue) (TGEV)	Coronaviridae
P0C6Y5	rep 1a-1b	Porcine transmissible gastroenteritis coronavirus (strain Purdue) (TGEV)	Coronaviridae
P0C6T4	1a	Bat coronavirus HKU4 (BtCoV) (BtCoV/HKU4/2004)	Coronaviridae
O12000		Rat cytomegalovirus (strain Maastricht)	Herpesviridae
Q2YHF2		Dengue virus type 4 (strain Thailand/0476/1997) (DENV-4)	Flaviviridae
Q83207		Murid herpesvirus 1 (strain Smith) (MuHV-1) (Mouse cytomegalovirus)	Herpesviridae
Q2YHF0		Dengue virus type 4 (strain Thailand/0348/1991) (DENV-4)	Flaviviridae
P09866		Dengue virus type 4 (strain Dominica/814669/1981) (DENV-4)	Flaviviridae
Q65165	Ba71V-075 B438L	African swine fever virus (strain Badajoz 1971 Vero-adapted) (Ba71V) (ASFV)	Asfarviridae
Q5UCB8		Dengue virus type 4 (strain Singapore/8976/1995) (DENV-4)	Flaviviridae
Q58HT7		Dengue virus type 4 (strain Philippines/H241/1956) (DENV-4)	Flaviviridae
Q8JJX1		Salmon pancreas disease virus (SPDV)	Togaviridae
Q8QL53		Sleeping disease virus (SDV)	Togaviridae

Q01035	74 ECRF3	Saimiriine herpesvirus 2 (strain 11) (SaHV-2) (Herpesvirus saimiri)	Herpesviridae
P08557	N Mup43	Enterobacteria phage Mu (Bacteriophage Mu)	Myoviridae (phages with contractile tails)
P24025	S	Hepatitis B virus genotype D (isolate France/alpha1/1989) (HBV-D)	Hepadnaviridae
Q18LE5		Elephantid herpesvirus 1 (isolate Asian elephant/Berlin/Kiba/1998) (EIHV-1) (Elephant endotheliotropic herpesvirus)	Herpesviridae
P21060	A37R	Vaccinia virus (strain Copenhagen) (VACV)	Poxviridae
P24762	VACWR160 A37R	Vaccinia virus (strain Western Reserve) (VACV) (Vaccinia virus (strain WR))	Poxviridae
Q84424	A103R	Paramecium bursaria Chlorella virus 1 (PBCV-1)	Phycodnaviridae
Q5UNV7	MIMI_L698	Acanthamoeba polyphaga mimivirus (APMV)	Mimiviridae
P13897		Western equine encephalitis virus (WEEV)	Togaviridae
Q8JJX0		Salmon pancreas disease virus (SPDV)	Togaviridae
Q8QL52		Sleeping disease virus (SDV)	Togaviridae
P16849	UL33	Human cytomegalovirus (strain AD169) (HHV-5) (Human herpesvirus 5)	Herpesviridae
Q6SW98	UL33	Human cytomegalovirus (strain Merlin) (HHV-5) (Human herpesvirus 5)	Herpesviridae
P25212	VACWR197 B15R	Vaccinia virus (strain Western Reserve) (VACV) (Vaccinia virus (strain WR))	Poxviridae
O57261	MVA184R ACAM3000_MVA_184	Vaccinia virus (strain Ankara) (VACV)	Poxviridae
P21116	B16R	Vaccinia virus (strain Copenhagen) (VACV)	Poxviridae
Q04523	CPXV209 B15R	Cowpox virus (strain Brighton Red) (CPV)	Poxviridae
Q37893	12	Bacillus phage B103 (Bacteriophage B103)	Podoviridae (phages with short tails)
P20747	10	Fowl adenovirus A serotype 1 (strain CELO / Phelps) (FAdV-1)	Adenoviridae

		(Avian adenovirus gal1 (strain Phelps))	
P31792	pol	Feline endogenous virus ECE1	Retroviridae
Q06998	S2	Bluetongue virus 3 (isolate South Africa vaccine) (BTV 3)	Reoviridae
P89466	UL46	Human herpesvirus 2 (strain HG52) (HHV-2) (Human herpes simplex virus 2)	Herpesviridae
P52463	U36 XIRF1	Human herpesvirus 6A (strain Uganda-1102) (HHV-6 variant A) (Human B lymphotropic virus)	Herpesviridae
Q9QJ33	U36	Human herpesvirus 6B (strain Z29) (HHV-6 variant B) (Human B lymphotropic virus)	Herpesviridae
P68677	E7	Equine herpesvirus 2 (strain T400/3) (EHV-2)	Herpesviridae
P68678	E7	Equine herpesvirus 2 (strain 86/87) (EHV-2)	Herpesviridae
P0CAP9	BCRF1	Epstein-Barr virus (strain GD1) (HHV-4) (Human herpesvirus 4)	Herpesviridae
P03180	BCRF1	Epstein-Barr virus (strain B95-8) (HHV-4) (Human herpesvirus 4)	Herpesviridae
P0C6Z6	BCRF1	Epstein-Barr virus (strain AG876) (HHV-4) (Human herpesvirus 4)	Herpesviridae
Q8JJE2	S9	Aquareovirus C (isolate Golden shiner/USA/GSRV/1977) (AQRV-C)	Reoviridae
P0C6T6	1a	Bat coronavirus HKU9 (BtCoV) (BtCoV/HKU9)	Coronaviridae
P0C6W5	rep 1a-1b	Bat coronavirus HKU9 (BtCoV) (BtCoV/HKU9)	Coronaviridae
P16472	TOP1 s074R	Rabbit fibroma virus (strain Kasza) (RFV) (Shope fibroma virus (strain Kasza))	Poxviridae
P24916	13 KCLF2	Saimiriine herpesvirus 2 (strain 11) (SaHV-2) (Herpesvirus saimiri)	Herpesviridae
O40633	13	Saimiriine herpesvirus 2 (strain 488) (SaHV-2) (Herpesvirus saimiri)	Herpesviridae
F5HAW0	ORF60	Human herpesvirus 8 type P (isolate GK18) (HHV-8) (Kaposi's sarcoma-associated herpesvirus)	Herpesviridae
P30318	POL LdOrf-83	Lymantria dispar multicapsid nuclear polyhedrosis virus (LdMNPV)	Baculoviridae

P31332	L	Sonchus yellow net virus (SYNV)	Rhabdoviridae
B2BNE1	S3	Aquareovirus G (isolate American grass carp/USA/PB01-155/-) (AQRV-G)	Reoviridae
